# Longitudinal effects of physical activity on sleep in association with hippocampal‐amygdala subfield structure and functional connectivity in Alzheimer's disease

**DOI:** 10.1002/alz.71651

**Published:** 2026-08-25

**Authors:** Ravi Dadsena, Ana S. Costa, Shari David, Luisa Haberl, Jörg B. Schulz, Kathrin Reetz, Alexa Haeger

**Affiliations:** ^1^ Centre for Dementia and Prevention Aachen (ZDPA) Department of Neurology RWTH Aachen University Aachen Germany; ^2^ JARA Brain Institute Translational Neuroscience Research Centre Jülich and RWTH Aachen University Aachen Germany

**Keywords:** Alzheimer's disease, cognitive decline, functional connectivity, medial temporal lobe subfields, neurodegeneration, neuroimaging, physical activity intervention, resting state, sleep

## Abstract

**INTRODUCTION:**

Sleep is a modifiable risk factor in Alzheimer's disease (AD), but its relationship to medial temporal lobe (MTL) subfields and responsiveness to lifestyle interventions remain unclear.

**METHODS:**

In the Dementia‐Multi‐Objective Validation of Exercise (MOVE) study, 46 individuals on the AD continuum were randomized to 6 months of multimodal exercise (*n* = 25) or psychoeducation (*n* = 21) and followed for 18 months (T1–T4). Clinical and cognitive testing, sleep/activity measures (PSQI, diaries, wearables), high‐resolution T1, and resting‐state functional magnetic resonance imaging scans were acquired. Hippocampal and amygdala subfield volumes and hippocampus−amygdala connectivity were analyzed longitudinally.

**RESULTS:**

After the 6‐month intervention, the intervention group showed increased hippocampus–amygdala connectivity and attenuated subfield atrophy. One year later, both groups progressed in cognitive decline and hippocampal atrophy. Hippocampal and amygdalar volumes and connectivity were associated with sleep parameters as well as activity and cognitive parameters.

**DISCUSSION:**

Exercise was associated with short‐term MTL preservation and network reorganization linked to sleep and activity, supporting continuous physical activity for lasting benefits.

## Highlights

- A 6‐month exercise intervention transiently preserved hippocampal‐amygdalar volumes.
- The intervention improved hippocampus‐amygdala connectivity during the active phase.
- Multimodal sleep assessment relates to MTL structure and connectivity.
- Findings link physical activity and sleep to MTL integrity and cognition in AD.
- Long‐term benefits require sustained intervention; disease progression resumes later.

## 1 BACKGROUND

Alzheimer's disease (AD) and related dementias (ADRD) represent a growing public health challenge. Chronic sleep disturbances and reduced sleep duration, common in the elderly,^1^ have been shown to increase dementia risk,^2^, ^3^ making sleep an important prevention target for cognitive decline.^4^, ^5^ Sleep facilitates glymphatic clearance of neurotoxic proteins such as amyloid beta (Aβ) and modulates synaptic plasticity, thereby supporting neural homeostasis and brain health.^6^, ^7^ Disrupted sleep architecture, particularly reductions in slow‐wave sleep, has been associated with cognitive decline and increased dementia risk, while disorders of rapid eye movement (REM) sleep may represent prodromal manifestations of neurodegenerative diseases.^5^, ^8^, ^9^

The medial temporal lobe (MTL), encompassing the hippocampus and amygdala, seems especially vulnerable to sleep disturbances and AD pathology: Hippocampal subfields are strongly linked to memory decline and sleep‐dependent plasticity. Amygdala nuclei are not only highly vulnerable to AD‐related neurodegeneration, as one of the centrally affected brain regions, but also play a central role in sleep‐sensitive limbic and emotional regulation and neuropsychiatric symptoms, which occur at high prevalence in AD.^10^, ^11^, ^12^, ^13^ In this context, the basolateral, centromedial, and superficial amygdala subdivisions that are also relevant for emotional arousal, stress reactivity, and affective symptoms seem to be frequently associated with disturbed sleep.^14^, ^15^ Amygdalar substructures are further implicated in REM‐sleep regulation,^16^, ^17^ making the amygdala relevant for sleep‐related research in neurodegeneration.^18^, ^19^ Recent neuroimaging studies have demonstrated that poor sleep quality is associated with increased hippocampal volume loss in cognitively normal older adults.^20^, ^21^ High‐resolution magnetic resonance imaging (MRI) segmentation can quantify hippocampal and amygdalar subregional volumes, offering enhanced sensitivity for detecting early pathological changes compared to global MTL measurements.^19^, ^22^

Beyond structural integrity, functional connectivity within MTL circuits appears disrupted in individuals with cognitive impairment, with altered hippocampus‐amygdala connectivity patterns in amnestic mild cognitive impairment.^23^, ^24^, ^25^ Resting‐state functional MRI (rs‐fMRI) can characterize these network‐level alterations and how sleep‐related factors may influence neural circuit function alongside structural decline.^23^ The bidirectional relationship between sleep disturbances and MTL degeneration suggests that poor sleep may contribute to neurodegeneration, while progressing neurodegeneration may further worsen sleep.^2^, ^5^ Emerging work also indicates that sleep fragmentation, irregular timing, and reduced sleep efficiency may affect hippocampus–amygdala limbic circuits, underscoring the importance of considering multiple sleep dimensions beyond duration.^8^, ^20^, ^26^, ^27^ Circadian disruption and misalignment have been linked to amyloid burden and cognitive decline, suggesting that sleep continuity and timing are relevant prevention targets.^3^, ^4^

Behavioral interventions targeting modifiable lifestyle factors represent promising strategies to mitigate AD risk.^28^, ^29^ Cognitive behavioral therapy for insomnia, psychoeducational programs, and multicomponent lifestyle interventions can improve sleep quality and cognitive outcomes in middle‐aged and older adults.^28^, ^30^ However, few studies have simultaneously examined structural and functional neural changes in MTL subregions as mechanistic pathways linking sleep improvement to sleep‐dependent memory consolidation and cognitive preservation.^31^, ^32^ Both the hippocampus and amygdala seem to be especially responsive to exercise, making these regions in association with their role in memory impairment, neurodegeneration, and sleep especially important for analyzing intervention‐mediated effects.^33^, ^34^, ^35^ Understanding whether behavioral interventions can modify sleep and sleep‐related MTL vulnerability at the subfield level provides insight into neuroprotective mechanisms and prevention strategies in AD.^19^, ^36^, ^37^ In particular, hippocampal subfields, for example CA1 and subiculum, together with amygdala nuclei may help characterize sleep‐related vulnerability and plasticity within a broader MTL‐limbic network rather than the hippocampus alone.^18^, ^19^

This study is based on the Dementia‐Multi‐Objective Validation of Exercise (MOVE) study,^38^ a randomized controlled trial examining the effect of a 6‐month multicomponent exercise program on cognitive and neural outcomes in patients with AD.^38^ Here, we investigated how physical activity influenced quality, duration, and fragmentation of sleep and how these related to hippocampal subfield and amygdala nucleus volumetry and functional connectivity over 18 months in the physical intervention compared to the psychoeducation control group. We tested whether the Dementia‐MOVE intervention differentially impacted sleep‐related MTL vulnerability and whether physical activity could influence the sleep–brain axis by preserving subfield volumes and stabilizing hippocampus–amygdala connectivity. This subfield‐resolved approach may advance sleep as a modifiable intervention target and inform neuroimaging marker development for AD prevention trials.

**RESEARCH IN CONTEXT**

1. **Systematic review**: We searched PubMed and Scopus for studies of exercise interventions in aging and AD/MCI examining sleep, cognition, and brain‐related outcomes. Most prior studies were single modality or cross‐sectional. Few included well‐characterized cohorts. Meta‐analyses suggest modest cognitive gains with exercise in AD, and imaging studies indicate that connectivity changes precede structural effects. However, evidence linking sleep, exercise, cognition, and brain imaging in the AD continuum remains limited.
2. **Interpretation**: In this randomized cohort with early AD, a 6‐month multicomponent exercise program was associated with higher hippocampus‐amygdala functional connectivity, preservation of structural volume and stronger associations among sleep, fitness, and cognition during the intervention, although disease progression was observed on long‐term follow‐up. These findings support the value of sustained physical activity as a prevention strategy in AD.
3. **Future directions**: Connectivity‐based markers, together with standardized fitness, sleep, brain metrics, and cognitive endpoints, should be integrated into longer‐term trials and their complex interplay should be considered. Clinical recommendations should include sustained physical activity.

## 2 METHODS

### 2.1 Study participants

The Dementia‐MOVE intervention trial was conducted at the Department of Neurology at the University Hospital RWTH Aachen, Germany. A total of 46 participants from the Alzheimer's continuum^39^ were initially enrolled, with 25 assigned to the intervention group and 21 to the psychoeducation control group. The screening and enrollment flow was previously reported in our published work^40^: 303 individuals were initially assessed for eligibility, 159 were excluded for not meeting the inclusion criteria, and 98 declined participation, resulting in a baseline sample of 46 participants.^40^ The sample size was based on sample sizes reported from previous intervention studies^41^, ^42^, ^43^, ^44^, ^45^ and based on power analyses related to the primary outcome of the trial (metabolic MRI), which was reported in the Dementia‐MOVE protocol^38^ and resulted in an estimated requirement of 22 participants per group. No separate a priori power calculation was performed for the present structural, functional connectivity, and brain‐behavior analyses. Randomization for group allocation was performed with WINPEPI software, applying age and fitness level, assessed via VO2max or the Six‐Minute Walk Test (6MWT) distance, as stratification factors, but not clinical staging. In case of odd numbers, stratification was performed in favor of the intervention group. The intervention took place in the physiotherapy department at the University Hospital RWTH Aachen or at a physiotherapy and medical training center in Simmerath, depending on the place of residence of the study participant. Protocols were uniformized between sites. Participants underwent outcome assessments at four time points: baseline (time point T_1_), 3 months (time point T_2_), 6 months directly after intervention (time point T_3_), and 18 months after baseline (time point T_4_, 12 months after the end of the 6‐month study period) at University Hospital RWTH Aachen. Structural MRI data were collected at three time points (T_1_, T_3_, and T_4_); rs‐fMRI data were collected at T_1_ and T_3_, with no functional data obtained at T_4_ due to a shortened MRI protocol. Eligible participants were between 50 and 80 years old and had a diagnosis of mild cognitive impairment (MCI) or mild dementia, fulfilling both clinical and biomarker criteria of AD alongside the AD continuum according to the AT(N) National Institute on Aging and Alzheimer's Association (NIA‐AA) criteria.^39^ In the total sample of 46 participants, 20 had a Clinical Dementia Rating (CDR) score of ≤0.5 (*n* = 17 for CDR = 0.5; *n* = 3 for CDR = 0), while 26 participants had a CDR score of ≥1.0 (*n* = 25 for CDR = 1.0, *n* = 1 for CDR = 2). The cerebrospinal (CSF) biomarker profile included Aβ1‐42 or the Aβ42/40 ratio (A), phosphorylated tau (T), and total tau (N), ensuring that all participants exhibited pathological amyloid accumulation (A^+^), with further stratification into A+T+ or A+T− subgroups. Thirty‐two participants fulfilled the complete A+T+N+ profile, nine participants were classified as A+T−, and one participant was classified as A+T+. In cases where CSF biomarkers were unavailable (*n* = 4), the diagnosis was based on neuroimaging and neuropsychological findings and expert clinical consensus according to the national dementia guidelines.^46^ Two of these subjects without CSF were included in the final sample. To assess whether inclusion of these participants influenced the results, we performed an additional sensitivity analysis excluding the two participants without CSF biomarker confirmation. Also, for two subjects with diagnosed obstructive sleep apnea from the intervention group, we performed an additional sensitivity analysis as well. The study and all protocol modifications received approval from the Ethics Committee of the Medical Faculty RWTH Aachen (EK 306/18) and were conducted in accordance with the latest guidelines of the Declaration of Helsinki. Written informed consent was obtained from all participants before study enrollment and any protocol adjustments. The trial was registered at ClinicalTrials.gov (NCT03939286). Further details regarding the Dementia‐MOVE study design, as well as the inclusion and exclusion criteria, were previously published.^38^

### 2.2 Procedures

#### 2.2.1 Physical activity intervention and control condition

The 6‐month program consisted of supervised multimodal exercise sessions combining aerobic endurance, resistance, and coordination training. Weekly 60‐min sessions were delivered in small groups by trained exercise therapists following a standardized protocol. Participants were also instructed to perform a moderate‐ to high‐intensity home‐based training for at least 30 min/week, as well as stretching and toning exercises for at least 15 min twice per week, following current recommendations on repetitive physical exercise.^47^, ^48^ Attendance and adherence were closely monitored. In addition, participants in the intervention group also received monthly structured psychoeducational sessions. Participants randomized to the psychoeducation control group only attended the psychoeducational sessions. These were offered monthly and lasted approximately 1 h. The program was designed for older adults with cognitive impairment and was delivered in easy‐to‐understand language, with time for explanations and questions. Topics included diagnostic procedures and therapeutic options in dementia, cognitive training, lifestyle factors, depression, sleep, communication, and social‐medical aspects of dementia care, promoting disease awareness, coping strategies, and lifestyle modifications relevant to brain health. In addition, a separate session on nutrition was offered.

The psychoeducational program was offered to participants in both the intervention and control groups and was also open to the interested public, except for the separate nutrition session, which was reserved for study participants. This approach was conceptually informed by multidomain lifestyle intervention studies such as the FINGER study^28^ to provide an active control condition and to account for non‐specific effects such as social interaction, regular contact with study staff, and engagement in structured group activities. Further details on the intervention and the psychoeducation control conditions can be taken from the study protocol.^38^

#### 2.2.2 Clinical and neuropsychological assessments

In short, clinical assessment included blood draw at baseline and after intervention at T_3_, including analysis of the lipid metabolism with levels of cholesterol and leptin. Determination of apolipoprotein E status was performed at baseline. Disease‐related characteristics and demographic information were collected at baseline and updated if needed. Anthropometric measurements, fitness, and neuropsychological assessments were collected at all time points. Cognitive function was assessed using the Montreal Cognitive Assessment (MoCA) and a comprehensive neuropsychological test battery using parallel versions (see also Haeger et al.^38^ for the detailed protocol). For analyses, composite scores were calculated for memory, attention, verbal memory, and executive function. Details on these calculations can be derived from a previous publication.^38^, ^49^ From available neuropsychiatric and quality‐of‐life measures, we analyzed data from the Hospital Anxiety and Depression Scale (HADS) to assess depressive and anxiety symptoms.^50^

#### 2.2.3 Physical fitness and activity monitoring

Physical fitness and activity levels were assessed using a combination of performance‐based tests, self‐reported measures, and wearable device tracking. Physical fitness assessments included maximal oxygen consumption (VO2max), the 6MWT, handgrip strength in the dominant hand assessed via a Saehan® hand dynamometer, and balance performance via the MFT‐S3‐Check® system. Fitness assessment included the estimation of the VO2max according to Astrand‐cycle testing and, in case of an insufficient increase in heart rate at 5/6 min < 110/min, the VO2max estimation was derived from the 6MWT.^51^ Objective activity data were collected using Fitbit devices for a time period of about 2 weeks mid‐intervention, including step count, distance covered, metabolic equivalent of task (MET) score, and moderate to vigorous physical activity (MVPA). Daily physical activity was further monitored through participant diaries, including reported daily activity and the corresponding strain, as well as changes in concomitant medication. Participants further reported their weekly weight. The frequency and duration of reported activity days were summarized. Strain metrics captured central tendency, variability, and the proportion of high‐strain days. Weight metrics included weight trends over the entire intervention period. Medication use and symptom severity were summarized separately from daily diaries. The Physical Activity Questionnaire (PAQ‐50+) was additionally used to estimate MET scores and MVPA at each time point T_1_ to T_4_. Mean scores over the assessment period were calculated.^38^, ^40^, ^52^

#### 2.2.4 Sleep assessment

Sleep was assessed using three methods: (1) based on a questionnaire – the Pittsburgh Sleep Quality Index (PSQI),^53^ (2) based on diaries kept during the intervention, and (3) via wearable fitness trackers (FitBit). The PSQI is a self‐report questionnaire measuring sleep quality and sleep disturbances over the previous month. It comprises 19 self‐rated items, which are summarized into seven component scores: subjective sleep quality, sleep latency, sleep duration, habitual sleep efficiency, sleep disturbances, use of sleep medication, and daytime dysfunction. Each component is scored on a scale from 0 to 3, with higher scores indicating poorer sleep quality. The global PSQI score is calculated as the sum of the seven component scores (range 0 to 21), with higher values reflecting worse overall sleep quality. For example, for the sleep medication component, participants rate the frequency of sleep‐medication use during the previous 4 weeks on a four‐point scale: 0 = not during the past 4 weeks, 1 = less than once per week, 2 = once or twice per week, and 3 = three or more times per week. Thus, values and differences for this component, including Δ values, are expressed in PSQI component‐score units and do not reflect the number of medications taken. Missing data in the PSQI were handled according to predefined rules: Missing items were imputed using person‐mean imputation when no more than two items were missing. Component and global PSQI scores were calculated only when sufficient data were available according to these criteria. PSQI was acquired at all time points.

For the diary, daily sleep data were aggregated into longitudinal summary metrics for each participant and time point, using either two (0 to 3 and 4 to 6 months) or six approximately monthly time bins across 6 months. All metrics were calculated at the Subject × Time level using only diary entries within the respective time bin. Sleep metrics were derived from daily self‐reported sleep duration and included measures of central tendency and variability, counts of short (<7 h) and long (>9 h) sleep nights, weekday–weekend differences, social jetlag, and data completeness. All variables were calculated using available data only; if no valid observations were present within a given time bin, the corresponding metrics were coded as missing. The diary was kept by the participants for a maximum total of 27 weeks (24 weeks including optional 3‐week prolongation) of the main intervention. Wrist‐worn wearable fitness trackers (FitBit) were further distributed mid‐intervention for recording of activity data and sleep duration for a maximum period of 2 weeks. The wearable data were used to obtain objective reference measures of physical activity and sleep duration in the overall sample, to assess concordance with self‐reported measures, and to monitor compliance with the intervention. We previously reported a strong association between wearable‐derived physical activity and self‐reported diary activities.^52^ Similarly, the high correlation between wearable‐derived objective and self‐reported subjective sleep assessments in this study (Figure S1) supports the use of all three sleep assessments.

### 2.3 MRI data acquisition

MRI data acquisition was conducted using a Siemens PRISMA 3T whole‐body scanner (Erlangen, Germany). High‐resolution T1‐weighted structural images and resting‐state functional scans were obtained. Structural imaging was performed using a T1‐weighted sequence with an isotropic voxel resolution of 0.8 mm (echo time [TE] = 2.36 ms, repetition time [TR] = 2.4 s, flip angle [FA] = 9°). Functional imaging was carried out using an echo‐planar imaging (EPI) sequence (matrix size = 64×64×36, 205 volumes/session, resolution = 3.1×3.1×3.6 mm^3^, TE = 30 ms, TR = 2.21 s, FA = 90°). To optimize rs‐fMRI acquisition, the scanning environment was adjusted to minimize ambient light. Participants were instructed to keep their eyes closed without engaging in specific thoughts and without sleeping, ensuring the capture of intrinsic brain activity in the absence of task‐related stimuli.

### 2.4 Processing of imaging data

The imaging data underwent a standardized preprocessing pipeline, starting with data organization in the Brain Imaging Data Structure (BIDS) format. To ensure high‐quality data for analysis, T1‐weighted anatomical and rs‐fMRI scans were subjected to a comprehensive quality control (QC) process using MRIQC (version 23.1.0).^54^ After the automated QC evaluation, all images were manually inspected for potential artifacts, ensuring their suitability for further processing (Text S1). For functional imaging, motion‐related artifacts were carefully addressed using predefined exclusion criteria. Mean framewise displacement (mFD) was calculated within each rs‐fMRI run across all acquired volumes. Scans were excluded from the functional analysis if (i) mFD exceeded 0.30 mm, (ii) more than 20% of frames showed FD > 0.2 mm, or (iii) any single frame showed FD > 5 mm.^55^ Only participants meeting the quality criteria entered subsequent analysis. For structural analysis, all participants were included. In the functional analysis, the final dataset comprised 23 participants in the intervention group at both baseline and at T_3_, while the psychoeducation control group included 19 participants at baseline and 16 at T_3_ (Table S1).

### 2.5 Structural MRI data

**FIGURE 1:**
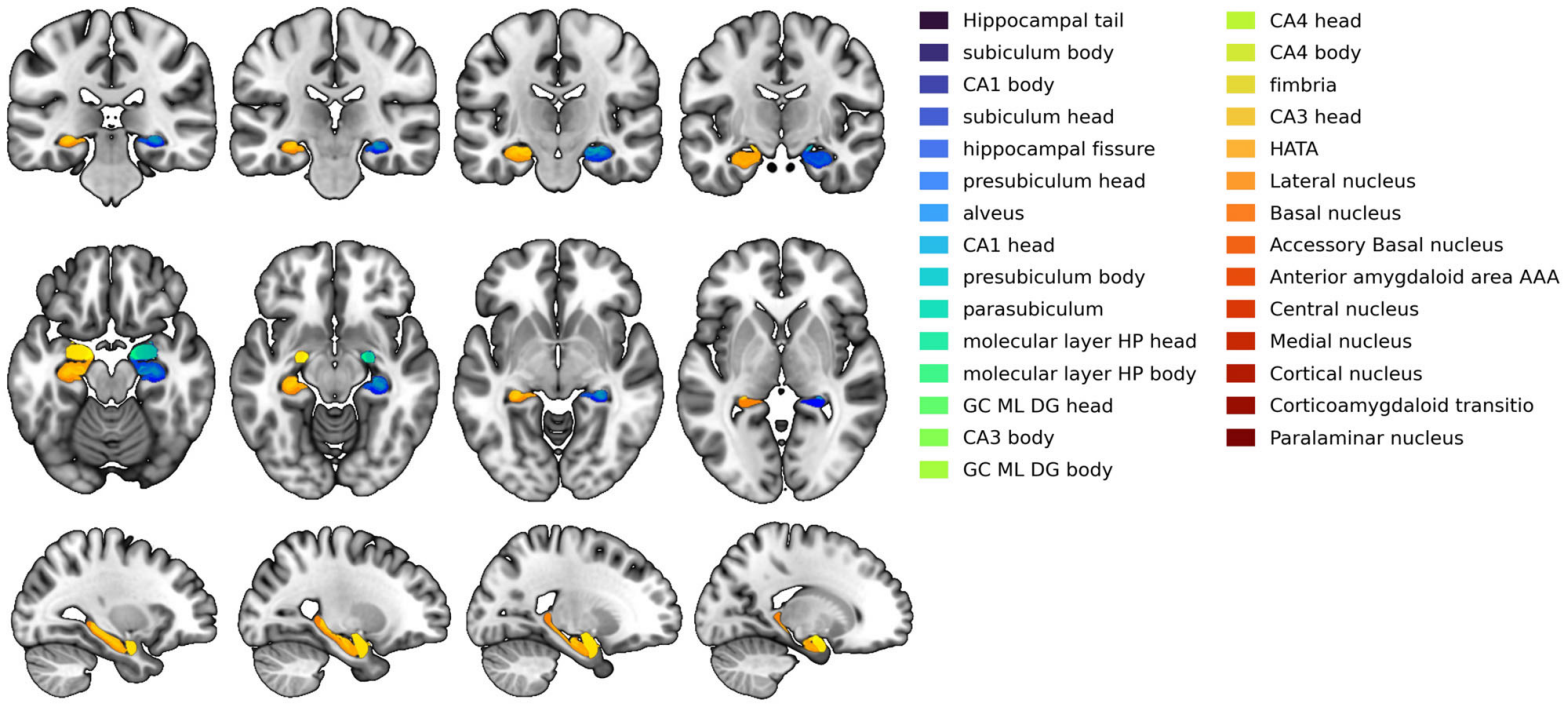
Hippocampal and amygdala subfield segmentations overlaid on MNI152 template brain, shown in representative coronal, axial, and sagittal slices to illustrate hippocampus–amygdala subfield anatomy. Color‐coded regions correspond to automatically segmented hippocampal and amygdala subfields derived from HippoAmyg atlas in MNI space and displayed as label overlays on the MNI152 template.

Structural MRI data were processed to derive subject‐specific volumes of hippocampal subfields and nuclei of the amygdala from T1‐weighted images using a two‐step pipeline. First, T1‐weighted images were processed with FastSurfer (version 2.3.0), a deep learning‐based structural processing framework that produces FreeSurfer‐compatible anatomical outputs and serves as the starting point for downstream segmentation. Building on these outputs, hippocampal subfields and amygdala nuclei were then segmented using FreeSurfer's (version 6.0) automated atlas‐based methods, which combine probabilistic anatomical atlases with subject‐specific image intensity information to delineate the internal subregional boundaries of the hippocampus and to subdivide the amygdala into anatomically defined nuclei (Figure 1).^22^, ^56^ The same standardized processing configuration was used for all sessions, followed by consistent visual quality control of the segmentations to assess anatomical accuracy. Longitudinal effects were evaluated using linear mixed‐effects models, with model details provided in the statistical analysis section. The resulting subregional volumes were extracted for each participant and used for subsequent statistical analyses, and total intracranial volume was included as a covariate in the relevant models to account for inter‐individual differences in head size.

### 2.6 Functional MRI data

The rs‐fMRI data were preprocessed using the fMRIPrep pipeline (version 23.2.3) following previously published work (Text S2).^57^ Briefly, fMRIPrep preprocessing included head‐motion correction, boundary‐based co‐registration of blood oxygen level‐dependent (BOLD) to T1w images, spatial normalization, and EPI susceptibility distortion correction using fMRIPrep's fieldmapless SyN approach. After preprocessing, denoising and spatial smoothing were performed using the CONN toolbox (version conn22v2407) using custom MATLAB scripts.^58^, ^59^ To eliminate potential confounding influences, several nuisance signals identified during preprocessing were regressed out. These included five principal components extracted from both white matter and CSF, session‐related effects along with their first‐order derivatives (two components), and general signal trends (three components). The resulting BOLD time series were then subjected to band‐pass filtering between 0.008 and 0.09 Hz to retain low‐frequency BOLD fluctuations relevant to resting‐state functional connectivity while attenuating slower signal drifts and higher‐frequency noise.^60^ Lastly, spatial smoothing was applied with an 8‐mm full‐width at half‐maximum Gaussian kernel to enhance signal clarity and facilitate better comparability across participants.^58^

Using these preprocessed data, we implemented region of interest (ROI)‐to‐ROI analyses focusing on connectivity between hippocampal and amygdalar subfields using the hippocampus–amygdala subfield atlas as the only set of ROIs. For each subfield, mean BOLD time series were extracted from the preprocessed and denoised rs‐fMRI data, and pairwise temporal correlations between all subfield time series were computed to generate subject‐specific connectivity matrices. Given the spatial resolution of the rs‐fMRI acquisition, these connectivity estimates were interpreted as atlas‐defined ROI‐level measures rather than fine‐grained voxel‐level subfield‐specific signals.^25^, ^61^ These matrices were then used to investigate group and time effects in subsequent statistical analyses.

### 2.7 Statistical analysis

To characterize longitudinal effects and connectivity alterations, we implemented an integrated analysis framework combining structural, functional, and clinical measures. Longitudinal associations were modeled using linear mixed‐effects (LME) models, which accommodate repeated measurements and incompletely observed data.^62^ All models were adjusted for age and sex to reduce potential confounding. Multiple testing correction was applied according to analysis type. For structural MRI measures and Spearman correlation analyses, *p* values were corrected using the Benjamini‐Hochberg false discovery rate (FDR) procedure. For ROI‐to‐ROI functional connectivity analyses, Holm‐Bonferroni correction was used as the primary correction method because these analyses involved a defined family of pairwise ROI tests. Results are presented with the correction method specified for each analysis type, with statistical significance evaluated at adjusted *p* < 0.05 for the corresponding correction method. In addition, given the high dimensionality of the imaging and connectivity data and the limited sample size, we applied a more stringent criterion for uncorrected effects, reporting only results with uncorrected *p* < 0.01 as exploratory findings. This strategy balances control of type I error with sensitivity in the presence of extensive multiple comparisons and follows common practice in neuroimaging studies. Structural data analyses were performed in R (version 4.3.1), and group‐level functional connectivity analyses were conducted with the CONN toolbox in MATLAB (version R2022a). For both structural measures and functional connectivity, LME models included group (intervention vs psychoeducation control), time, and their interaction as fixed effects, with subject‐specific random intercepts to model within‐subject correlations across time points. Moreover, Spearman's rank correlations were conducted to assess associations among structural, functional, and clinical variables, with FDR correction applied across the tested correlations. These correlations were exploratory, with interpretation focused on corrected results and consistent patterns across related sleep, clinical, and hippocampus–amygdala measures. Both uncorrected and corrected *p* values are presented, where uncorrected results are considered exploratory and corrected values guide the primary conclusions.^63^

## 3 RESULTS

### 3.1 Demographic and clinical characteristics of sample and clinical changes over time

At baseline, there was no significant difference between both groups in most of the assessed parameters; however, there was a tendency toward group differences in cognitive performance on MoCA, verbal memory composite, executive function composite, neurodegenerative markers from CSF, and cardiovascular risk profile after correction for multiple comparisons (Table 1). The demographics for the adapted sample included for the functional and structural MRI analysis can be found in Table S1.

**TABLE 1:** Baseline demographic and clinical characteristics of intervention and psychoeducation control groups. Lab‐specific AD‐relevant cut‐off values for cerebrospinal fluid parameters are as follows: Aβ 1‐42 < 450 pg/mL, Aβ 42/40‐ratio < 0.5, tau > 450 pg/mL, phospho‐tau > 61 pg/mL, analysis was performed in the Neurochemical Laboratory at the University of Göttingen.^64^, ^65^

	Total			Intervention			Psychoeducation			
Characteristics at baseline	Mean	SD	*n*	Mean	SD	*n*	Mean	SD	*n*	*p* value (FDR‐corr.)
**Demographic characteristics**										
Age at baseline (years)	70.7	7.0	46	71.6	5.8	25	68.6	8.0	21	0.31
Sex (% female)	39		46	40		25	38		21	0.90
Education (years)	13.3	3.3	46	12.2	3.7	25	14.6	2.4	21	0.056
Disease duration (years)	3.7	1.9	46	3.8	2.1	25	3.5	1.7	21	0.66
Clinical Dementia Rating (CDR)	0.8	0.4	46	0.9	0.3	25	0.7	0.4	21	0.50
**Apolipoprotein E4 status**										
APOE4 (% APOE4 heterozygotes/homozygotes)	54/17		46	44/24		25	67/10		21	0.51
**Cerebrospinal fluid biomarkers**										
Aβ1‐40 (pg/mL)	10485.1	4122.3	40	11083.6	3571.5	21	9823.6	4665	19	0.48
Aβ1‐42 (pg/mL)	410.7	165.1	41	393.8	162.9	21	428.4	169.7	20	0.57
Aβ1‐42/Aβ1‐40 ratio	0.498	0.58	40	0.36	0.10	21	0.65	0.82	19	0.27
Phospho‐tau (pg/mL)	85.6	36.3	42	95.3	35.9	21	76.0	34.9	21	0.23
Total tau (pg/mL)	598.7	318.1	42	709.0	317.8	21	488.5	284.5	21	0.08
**Anthropometric measurements**										
Body mass index (kg/m^2^)	25	4	46	25	5	25	24	3	21	0.73
Fat mass (%)	25	9	46	24	9	25	25	9	21	0.75
Muscle mass (%)	32	4	45	32	5	25	32	4	20	0.75
Waist‐to‐hip ratio	0.91	0.081	46	0.91	0.081	25	0.91	0.084	21	0.74
**Fitness**										
VO2max (mL/min/kg)	28.6	5.5	46	28.3	5.2	25	28.8	5.9	21	0.72
6‐minute‐walk distance (m)	529.3	91.5	46	528.6	101.6	25	530.2	80.3	21	0.71
Handgrip strength	30.5	9.7	46	30.7	9.6	25	30.4	10.1	21	0.71
Balance composite score	−0.07	0.56	45	−0.11	0.53	24	−0.02	0.6	21	0.62
**Activity parameters**										
Daily PAQ50+ MET‐hours	18.4	8.5	46	19.9	7.5	25	16.7	9.4	21	0.34
Daily Fitbit MET total average	5.7	2.5	41	5.3	2.3	24	6.4	2.7	17	0.31
Daily Fitbit step count (average steps per day)	9328	4190	41	8690	3776	24	10228	4683	17	0.46
Daily Fitbit moderate activity (average min per day)	32.2	26.9	41	29.8	22.7	24	35.6	32.2	17	0.73
Daily Fitbit vigorous activity (average min per day)	35.9	28.8	41	34.4	29.6	24	38.0	28.5	17	0.62
Daily diary moderate activity (min/day)	41.9	51.3	40	25.8	21.4	24	66.0	71.5	16	0.21
Daily diary MVPA (min/day)	67.0	50.4	40	53.0	33.6	24	88.1	64.0	16	0.2
**Cardiovascular risk profile**										
Hypertension (% yes)	61		46	80		25	38		21	0.067
Diabetes (% yes)	7		46	8		25	5		21	0.70
Active smoking (% yes)	4		46	0		25	10		21	0.49
Number of cardiovascular risk factors	1.5	0.8	46	1.8	0.8	25	1.3	0.7	21	0.12
**Neuropsychological performance**										
Mini‐Mental State Examination	24.28	3.32	46	23.92	3.12	25	24.71	3.57	21	0.51
Montreal Cognitive Assessment	18.91	4.69	46	17.16	3.85	25	21.00	4.83	21	0.13
Attention composite score	0.00	0.96	46	−0.17	1.03	25	0.20	0.86	21	0.33
Executive function composite score	−0.005	0.64	46	−0.22	0.60	25	0.25	0.60	21	0.053
Verbal memory composite score	0.00	0.49	46	−0.17	0.49	25	0.21	0.42	21	0.067
HADS (total score)	7.54	6.24	46	6.44	5.56	25	8.86	6.87	21	0.35
Pittsburgh Sleep Quality Index	4.80	3.48	46	4.20	3.32	25	5.52	3.61	21	0.33

*Mean values and standard deviations (SD) are presented for the whole group. Composite scores represent z‐scores. Reported *p* values are FDR‐corrected. Daily PAQ50+ MET‐hours were calculated according to Ainsworth et al., 2000. Daily Fitbit MET total average was estimated from daily walking distance using the following formula: $\bar{\mathrm{MET}\;(h/d)}=\frac{1}{n}\sum_{d=1}^{n}\left(\frac{D_{d}}{4}\times 3.5\right)$ assuming a standard walking speed of 4 km/h and a metabolic equivalent of task (MET) value of 3.5 for walking at a normal pace.^66^ The daily mean was calculated across all available Fitbit data.

Over the intervention period (T_1_ to T_3_), the intervention group demonstrated significant within‐group improvements in cardiorespiratory fitness (VO_2_max), with a corresponding T_3_−T_1_ difference of +1.66 mL/kg/min (*p* = 0.0052). In the intervention group, there were additionally trend‐level improvements in stability and balance scores. For motor performance, the nominal Group × Time interaction for right handgrip strength was driven by an increase in the psychoeducation control group from T_1_ to T_3_ (Δ = +2.26; *p* = 0.013), while the intervention group showed no clear change. For cognition, there was no global effect of the intervention on cognition. The intervention group and the psychoeducation control group showed stable cognitive values both for MoCA and for the composite scores alongside the intervention duration. Depressive symptoms (HADS depression subscore) increased in both groups. No corresponding Group × Time interaction was observed (*p* = 0.48), indicating broadly similar time trends across arms (see also Table 3).

For sleep, within‐group time effects suggested decreased daytime tiredness in the intervention group over time. In the psychoeducation control group, there were indications of reduced sleep disturbance, reduced PSQI total sum, and reduced sleep medication use at T_2_ and T_3_ compared to baseline T_1_ in the psychoeducation control group. A baseline between‐group difference was observed for the PSQI medication component at T_1_ (Δ = −0.476; p = 0.0045), with lower reported sleep‐medication use in the intervention group at baseline. Subjective sleep quality (PSQI components) showed no Group × Time interactions.

Sleep reporting in the diaries over 27 weeks revealed significant increases in the count of long sleep nights (> 9 h) and in the maximal hours of sleep over time in the intervention group (Table 3). The psychoeducation control group also showed an increased number and rate of long sleep nights. Interestingly, the psychoeducation control group showed trends for increased social jetlag hours and weekend catchup and mean hours, whereas this was not the case for the intervention group. Overall, there was no significant Group × Time interaction on the sleep parameters from the diary. For reported physical activity, both groups showed increasing total activity over time, accompanied by increases in active‐day and inactive‐day counts in both arms, consistent with greater diary coverage over time. The active‐day percentage declined modestly in the intervention group but was stable in the psychoeducation control group, while mean minutes per active day did not change in either group. LME models showed no evidence for Group × Time interactions for any diary‐derived activity metric, indicating broadly similar trajectories between intervention and psychoeducation control. Physiological strain variability showed a significant Group × Time interaction: The intervention group exhibited significant within‐group increases in high‐strain day count and overall strain exposure, whereas the psychoeducation control group showed significant within‐group decreases in strain variability (standard deviation), percentage of high‐strain days, and maximum strain scores. Body weight trajectories from the diaries showed no significant Group × Time interaction. All results are reported with both uncorrected and FDR‐corrected *p* values in Table 2, Table 3, and Table S2. Regarding the association between activity and sleep recorded in the diaries, activity frequency was negatively correlated with social jetlag, pointing to more active days being associated with a smaller weekday–weekend discrepancy and more regular sleep timing. Higher activity levels (both total monthly hours and minutes per active day) were further associated with greater weekend catch‐up sleep and vice versa. Strain was further negatively associated with sleep duration, both affecting weekday and weekend mean hours.

**TABLE 2:** Diary‐based sleep, weight, activity, and strain measures over 27 weeks in the intervention and psychoeducation control groups.

	Total			Intervention			Psychoeducation			
Characteristics over the 27‐week monitoring period	Mean	SD	*n*	Mean	SD	*n*	Mean	SD	*n*	*p* value (FDR‐corr.)
**Sleep parameters derived from sleep diaries (average over 27** **weeks)**										
Mean sleep duration (h/night)	7.7	1.2	40	7.7	1.4	23	7.6	0.8	17	0.87
Long nights (% of nights)	10.5	21.3	40	14.7	25.1	23	4.8	13.5	17	0.35
Short nights (% of nights)	19.5	28.2	40	22.0	31.4	23	16.1	23.7	17	0.61
Sleep data completeness (%)	78.8	21.3	40	79.9	17.4	23	77.3	26.1	17	0.78
Sleep duration variability (CV %)	10.9	5.7	40	11.8	5.8	23	9.6	5.51	17	0.48
Sleep SD hours	0.8	0.3	40	0.8	0.3	23	0.7	0.3	17	0.40
Social jetlag (h)	0.2	0.2	40	0.2	0.2	23	0.2	0.2	17	0.68
**Monitoring parameters derived from sleep diaries (average over 27** **weeks)**										
Active days (% of days)	77.5	19.9	41	73.6	19.8	24	83.0	19.2	17	0.35
Minutes per active day (mean)	75.3	39.2	41	60.9	29.4	24	95.7	43.0	17	0.04
Total activity hours (27 weeks)	193.1	192.93	41	129.4	108.6	24	283.1	247.7	17	0.10
**Physiological strain derived from sleep diaries (average over 27** **weeks)**										
High‐strain days (% of days)	67.0	36.1	41	81.7	30.2	24	46.1	34.0	17	0.02
Mean strain score	4.1	2.1	41	4.8	2.0	24	3.1	1.8	17	0.04

*Mean values and standard deviations (SD) are presented. Composite scores represent z‐scores. Reported *p* values are FDR‐corrected. Sleep and activity measures reflect averages over the 27‐week monitoring period; all other variables represent baseline assessments. Definitions: Long nights (% of nights) = Percentage of nights with sleep duration > 9 hours; Short nights (% of nights) = Percentage of nights with sleep duration < 7 hours; Sleep data completeness (%) = data completeness in percent; Sleep SD hours = standard deviation (SD) of all nightly sleep durations; Sleep duration variability (CV %) = coefficient of variation in percent ((Sample standard deviation of all nightly sleep durations / mean of all nightly sleep durations)*100); Social jetlag (h) = absolute value of difference between the mean sleep hours on the weekend and the mean sleep hours on weekdays; High‐strain days (% of days) = Percentage of days with strain score >= 3.

**TABLE 3:** Changes over time in clinical and key self‐reported sleep and activity diary measures in intervention and psychoeducation control groups.

Clinical Measure	Group	Estimate	SE	*t*‐value	*p* value	*p* FDR direction
**Cognitive function**						
Attention composite score	Intervention	−0.05	0.08	−0.55	0.58	0.6
	Psychoeducation	−0.05	0.05	−0.79	0.43	0.77
Executive function composite score	Intervention	−0.04	0.03	−1.23	0.22	0.31
	Psychoeducation	0	0.03	0.01	0.98	0.99
Montreal Cognitive Assessment	Intervention	−1.23	0.29	−4.22	**0.001**	**0.004**
	Psychoeducation	−0.79	0.28	−2.76	**0.04**	0.12
Verbal memory composite score	Intervention	0.02	0.05	0.4	0.68	0.68
	Psychoeducation	0	0.05	0.04	0.96	0.99
**Physical activity**						
PAQ50+ calories daily	Intervention	−68.05	80.12	−0.85	0.39	0.52
	Psychoeducation	−113.8	118.56	−0.96	0.34	0.73
PAQ50+ met daily	Intervention	−0.84	1.06	−0.79	0.43	0.53
	Psychoeducation	−1.07	1.49	−0.71	0.48	0.8
PAQ50+ moderate daily	Intervention	−12.85	7.58	−1.7	0.09	0.2
	Psychoeducation	−0.87	11.01	−0.08	0.93	0.99
PAQ50+ MVPA daily	Intervention	−5.83	9.91	−0.59	0.55	0.59
	Psychoeducation	−7.51	13.13	−0.58	0.57	0.88
PAQ50+ vigorous daily	Intervention	7.07	4.77	1.48	0.14	0.24
	Psychoeducation	−7.2	5.31	−1.36	0.18	0.54
Daily energy expenditure	Intervention	−39.02	20.07	−1.95	**0.05**	0.16
	Psychoeducation	6.85	34.28	0.19	0.84	0.99
Daily metabolic equivalent of task	Intervention	−0.55	0.24	−2.19	**0.03**	0.12
	Psychoeducation	−0.01	0.5	−0.01	0.99	0.99
Daily moderate‐intensity physical activity	Intervention	−3.08	1.71	−1.8	0.07	0.18
	Psychoeducation	2.62	6.2	0.42	0.67	0.92
Daily moderate‐to‐vigorous physical activity	Intervention	−6.06	2.35	−2.57	**0.01**	0.08
	Psychoeducation	−0.91	6.41	−0.15	0.88	0.99
Daily vigorous‐intensity physical activity	Intervention	−3.01	2.18	−1.38	0.17	0.26
	Psychoeducation	−3.41	2.73	−1.25	0.22	0.55
**Physical Fitness**						
Balance symmetry difference (anteroposterior)	Intervention	1.01	1.61	0.62	0.53	0.59
	Psychoeducation	−2.86	1.9	−1.5	0.13	0.46
Balance symmetry difference (mediolateral)	Intervention	5.45	1.74	3.12	**0.002**	**0.03**
	Psychoeducation	1.32	1.68	0.78	0.43	0.77
Composite balance score	Intervention	0.05	0.03	1.38	0.17	0.26
	Psychoeducation	−0.05	0.03	−1.31	0.19	0.54
Handgrip strength (dominant hand)	Intervention	4.32	2.83	1.52	0.13	0.23
	Psychoeducation	0.68	0.31	2.16	**0.03**	0.26
Handgrip strength (left hand)	Intervention	−0.49	0.26	−1.86	0.06	0.17
	Psychoeducation	0.56	0.35	1.59	0.11	0.44
Handgrip strength (right hand)	Intervention	−0.42	0.26	−1.56	0.12	0.23
	Psychoeducation	0.95	0.3	3.09	**0.003**	0.09
Maximal oxygen uptake (VO_2_max)	Intervention	0.58	0.27	2.13	**0.005**	**0.04**
	Psychoeducation	0.41	0.24	1.7	0.33	0.64
Postural stability index (anteroposterior)	Intervention	−0.27	0.09	−2.97	**0.004**	**0.03**
	Psychoeducation	0.05	0.06	0.82	0.41	0.77
Postural stability index (mediolateral)	Intervention	−0.17	0.09	−1.66	0.1	0.2
	Psychoeducation	0.04	0.1	0.4	0.69	0.92
Sensorimotor balance index (anteroposterior)	Intervention	−0.29	0.12	−2.33	**0.02**	0.11
	Psychoeducation	0.18	0.11	1.64	0.1	0.44
Sensorimotor balance index (mediolateral)	Intervention	−0.4	0.12	−3.08	**0.002**	**0.03**
	Psychoeducation	−0.05	0.12	−0.38	0.71	0.92
Six‐minute walk test distance	Intervention	−7.88	3.7	−2.13	**0.03**	0.12
	Psychoeducation	8.23	5.09	1.61	0.11	0.44
**Psychological health**						
HADS Anxiety subscale	Intervention	−0.23	0.26	−0.85	0.4	0.52
	Psychoeducation	−0.2	0.35	−0.55	0.58	0.88
HADS Depression subscale	Intervention	0.5	0.26	1.89	**0.005**	**0.01**
	Psychoeducation	0.3	0.27	1.11	**0.02**	0.15
HADS Hospital Anxiety and Depression Scale (total)	Intervention	0.27	0.46	0.59	0.008	0.06
	Psychoeducation	0.1	0.52	0.2	0.16	0.48
Pittsburgh Sleep Quality Index	Intervention	0.2	0.26	0.77	0.44	0.53
	Psychoeducation	−0.77	0.32	−2.34	**0.02**	0.24
**Monitoring parameters (over 27** **weeks)**						
Activity active percent	Intervention	−1.94	0.91	−2.1	**0.03**	**0.04**
	Psychoeducation	−1.06	1.32	−0.8	0.42	0.42
Activity mean minutes per ActiveDay	Intervention	−0.36	0.98	−0.37	0.71	0.71
	Psychoeducation	2.93	2.21	1.32	0.18	0.22
Activity total minutes	Intervention	91.71	38.58	2.37	**0.01**	**0.02**
	Psychoeducation	318.12	106.73	2.98	**0.003**	**0.005**
**Physiological strain**						
Strain high days percent	Intervention	0.39	0.81	0.48	0.63	0.85
	Psychoeducation	−2.87	1.23	−2.33	**0.02**	**0.04**
Strain max score	Intervention	−0.02	0.07	−0.18	0.85	0.85
	Psychoeducation	−0.17	0.06	−2.38	**0.02**	**0.04**
Strain mean score	Intervention	0.06	0.06	1	0.31	0.73
	Psychoeducation	−0.1	0.05	−1.92	0.05	0.08
Strain SD score	Intervention	−0.01	0.02	−0.3	0.77	0.85
	Psychoeducation	−0.06	0.02	−2.31	**0.02**	**0.04**
**Sleep quality (over 27** **weeks)**						
Sleep CV percent	Intervention	0.07	0.21	0.36	0.71	0.78
	Psychoeducation	−0.12	0.16	−0.69	0.49	0.59
Sleep LongNights count	Intervention	1	0.46	2.17	**0.03**	0.2
	Psychoeducation	0.45	0.18	2.42	**0.01**	0.14
Sleep LongNights percent	Intervention	1.23	0.71	1.72	0.08	0.33
	Psychoeducation	1.11	0.48	2.3	**0.02**	0.14
Sleep mean hours	Intervention	0.03	0.02	1.28	0.2	0.38
	Psychoeducation	0.03	0.02	1.48	0.14	0.27
Sleep SD (h)	Intervention	0	0.01	0.48	0.63	0.78
	Psychoeducation	−0.01	0.01	−0.3	0.76	0.76
Sleep ShortNights count	Intervention	0.52	0.3	1.73	0.08	0.33
	Psychoeducation	0.42	0.27	1.54	0.12	0.27
Sleep ShortNights percent	Intervention	−0.28	0.78	−0.36	0.72	0.78
	Psychoeducation	−0.61	0.79	−0.77	0.44	0.56
Sleep social jetlag hours	Intervention	−0.03	0.01	−1.38	0.17	0.38
	Psychoeducation	0.03	0.01	1.74	0.08	0.23

*Note*: The table presents statistically significant changes over time in clinical and diary‐derived measures. Clinical domains (cognitive function, physical activity, physical fitness, psychological health) were obtained at four visits (T_1_– T_4_), whereas wearable‐derived domains (monitoring parameters, physiological strain, weight parameters, sleep quality) summarize data collected over 27 weeks.

Abbreviations: met, metabolic equivalent of task; MVPA, moderate‐to‐vigorous physical activity; sleep CV percent, coefficient of variation of nightly sleep duration in percentage; Sleep SD hours, sample standard deviation of all nightly sleep durations (hours).

### 3.2 Structural brain changes

Hippocampal and amygdalar subfield volumes were comparable between groups at baseline, with no statistically significant differences in overall measures. During the 6‐month intervention period (T_1_ to T_3_), no hippocampal or amygdalar subfield volume changes survived correction for multiple comparisons in either group; uncorrected exploratory effects are reported in Tables S3–S5. Over the 18‐month study period (T_1_ to T_4_), also including the 12‐month interval after the end of the intervention, both groups showed hippocampal volume loss, but this pattern was more pronounced in the intervention group. In this group, significant atrophy emerged in several right hippocampal subfields, including the subiculum head, presubiculum head, dentate gyrus body, CA4 body, and fimbria over the long term. Additional uncorrected exploratory volume reductions are reported in Tables S3–S5. The psychoeducation control group also exhibited small hippocampal volume declines over the total time period of 18 months, but none of these effects survived correction for multiple comparisons. No between‐group structural effects or Group × Time interactions survived correction for multiple comparisons.

For the amygdala, no amygdala subfield changes survived correction for multiple comparisons during the intervention period or over the full 18‐month period. Uncorrected exploratory effects are reported in Tables S3–S5. No statistically significant Group × Time interactions were identified for any amygdala subfield for the whole assessment period. All structural findings, including uncorrected and multiple‐comparison–corrected *p* values, are summarized in Tables S3–S5 and S6.

### 3.3 Functional brain changes

Hippocampal and amygdala subfield connectivity patterns already differed between groups at T_1_. The intervention group showed predominantly stronger connectivity in several right‐hemisphere hippocampal pathways, especially those involving the subiculum. From T_1_ to T_3_, both groups exhibited changes in hippocampus–amygdala connectivity, but the magnitude and extent of these changes differed. The intervention group showed increased connectivity strength in a larger number of connections than the psychoeducation control group. After correction for multiple comparisons, the pathway linking the left lateral amygdala nucleus to the right hippocampal presubiculum head remained, showing increased connectivity over this period. Significant connectivity changes were also observed in the psychoeducation control group. Across both groups, connectivity changes during this interval were predominantly increases.

**FIGURE 2:**
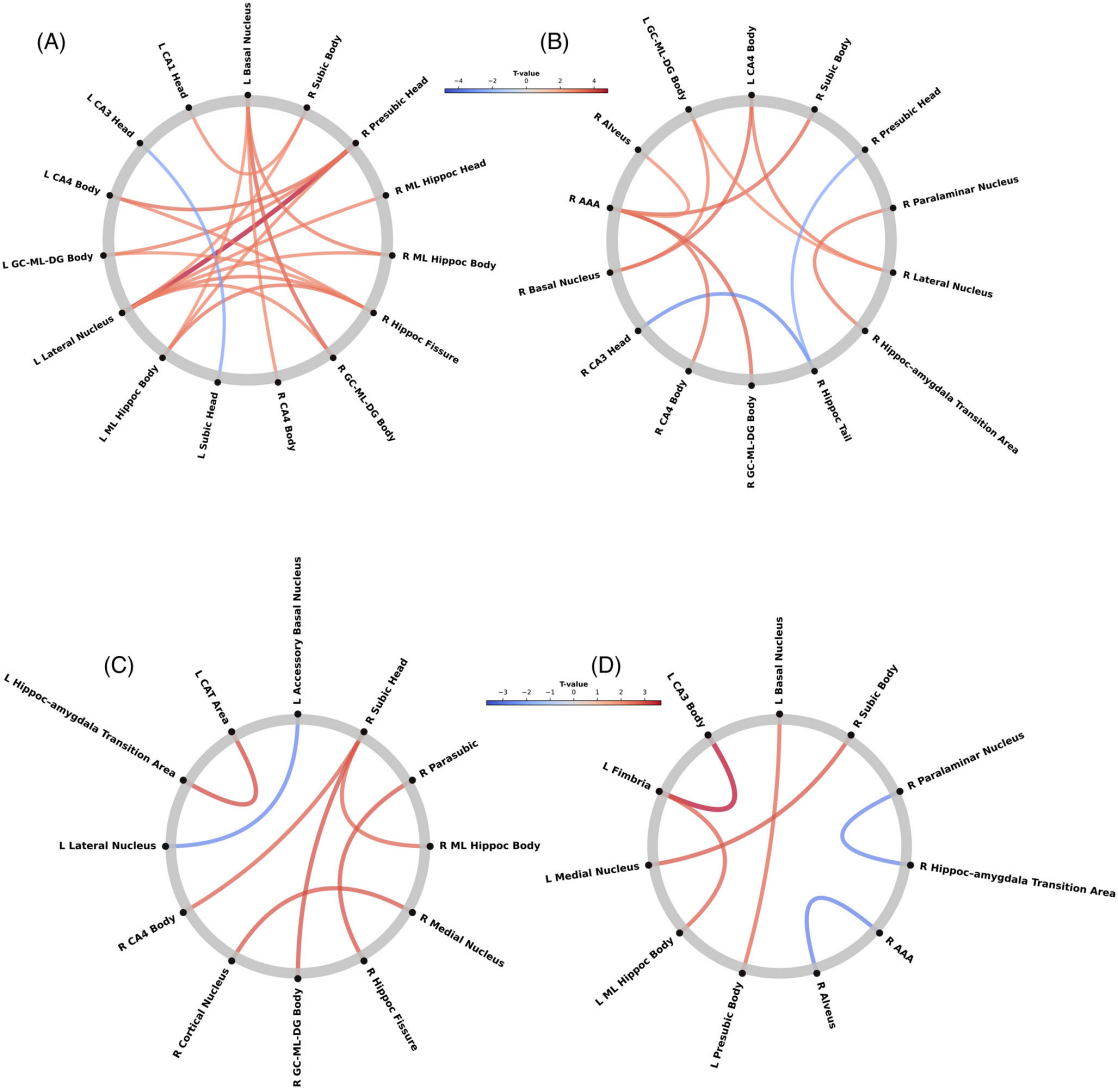
Resting‐state functional connectivity changes in hippocampal–amygdala subfield networks. (A) Intervention: change from T1 to T3. (B) Psychoeducation control: change from T1 to T3. (C) Between‐group difference at T1 (intervention – psychoeducation control). (D) Between‐group difference at T3 (intervention – psychoeducation control). Red indicates increased functional connectivity, whereas blue indicates decreased functional connectivity.

At T_3_, significant between‐group differences were still apparent in several connections. The intervention group showed stronger connectivity in hippocampal–fimbria pathways and in cross‐hemispheric subiculum–amygdala connections, whereas the psychoeducation control group showed stronger connectivity in specific amygdala subnuclei pathways. Group × Time interaction effects did not survive correction for multiple comparisons and are therefore not described as significant main findings. These functional connectivity findings are summarized in Figure 2, with detailed statistical results provided in Tables S7 and S8.

### 3.4 Correlations between longitudinal changes in brain measures and clinical variables

**FIGURE 3:**
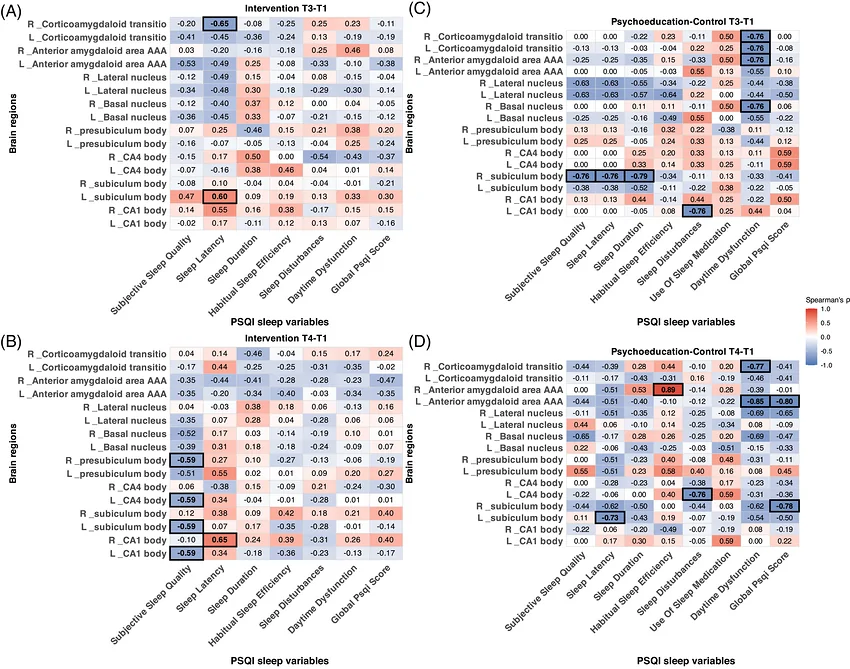
Correlation heatmaps illustrating associations between changes in PSQI sleep measures and longitudinal changes in hippocampal subfield and amygdala nucleus volumes across study groups and time intervals. (A) Intervention ΔT3–T1. (B) Intervention ΔT4–T1. (C) Psychoeducation control ΔT3–T1. (D) Psychoeducation control ΔT4–T1. Spearman's rank correlation was used to assess associations between change scores. Statistical significance was determined using false discovery rate (FDR) correction (*p* < 0.05), with bold values indicating significant correlations. Each cell in the heatmap represents a pairwise correlation between the change in a clinical marker and the corresponding change in a hippocampal subfield or amygdala nucleus volume.

Longitudinal changes in PSQI sleep measures were associated with longitudinal volume change across hippocampal subfields and amygdala nuclei across study groups and time intervals (Figure 3A–D and Figure S2). The dominant pattern comprised negative correlations, with moderate to strong associations concentrated in specific subfields and nuclei rather than uniformly across all regions. The most consistently associated PSQI dimensions were subjective sleep quality, sleep latency, daytime dysfunction, and global PSQI, with additional interval‐ and group‐dependent associations for habitual sleep efficiency (Figure 3). The magnitude and distribution of sleep–structure correlations varied across groups and intervals, with strong associations present in both groups (Figure 3A–D and Figure S2).

**FIGURE 4:**
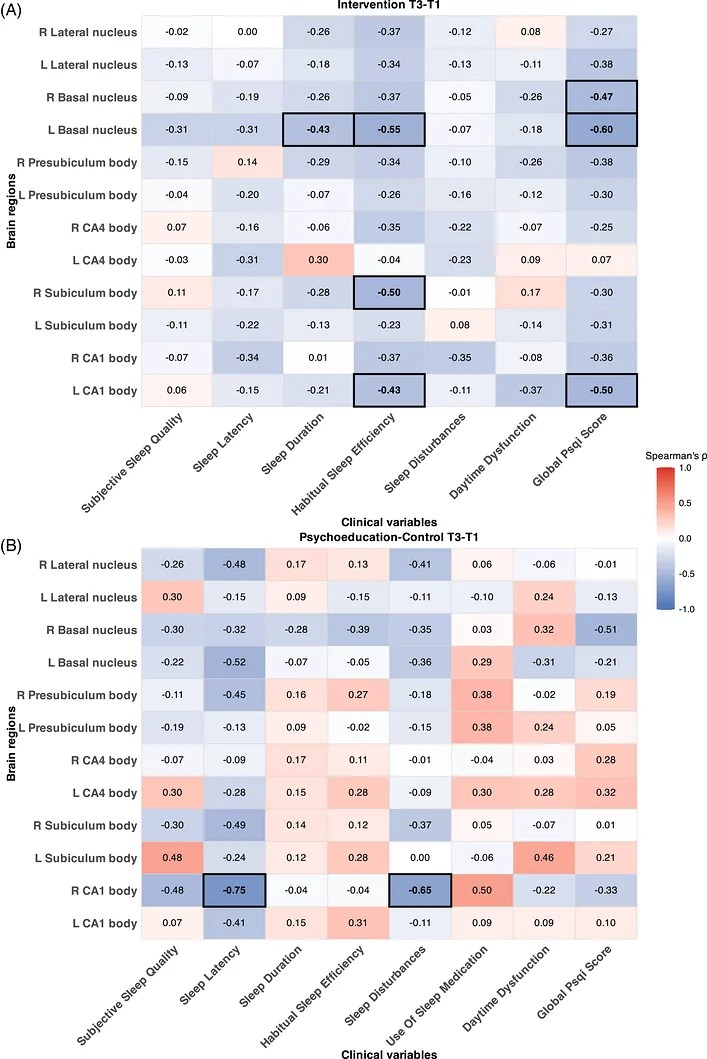
Correlation heatmaps illustrating associations between changes in PSQI sleep measures and longitudinal changes in functional connectivity within hippocampal subfield and amygdala nucleus networks across study groups for T_3_–T_1_ interval. (A) Intervention ΔT_3_– T_1_. (B) Psychoeducation control ΔT_3_–T_1_. Spearman's rank correlation was used to assess associations between change scores. Statistical significance was determined using false discovery rate (FDR) correction (*p* < 0.05), with bold values indicating significant correlations. Each cell in the heatmap represents a pairwise correlation between the change in a clinical marker and the corresponding change in a functional connectivity measure.

**FIGURE 5:**
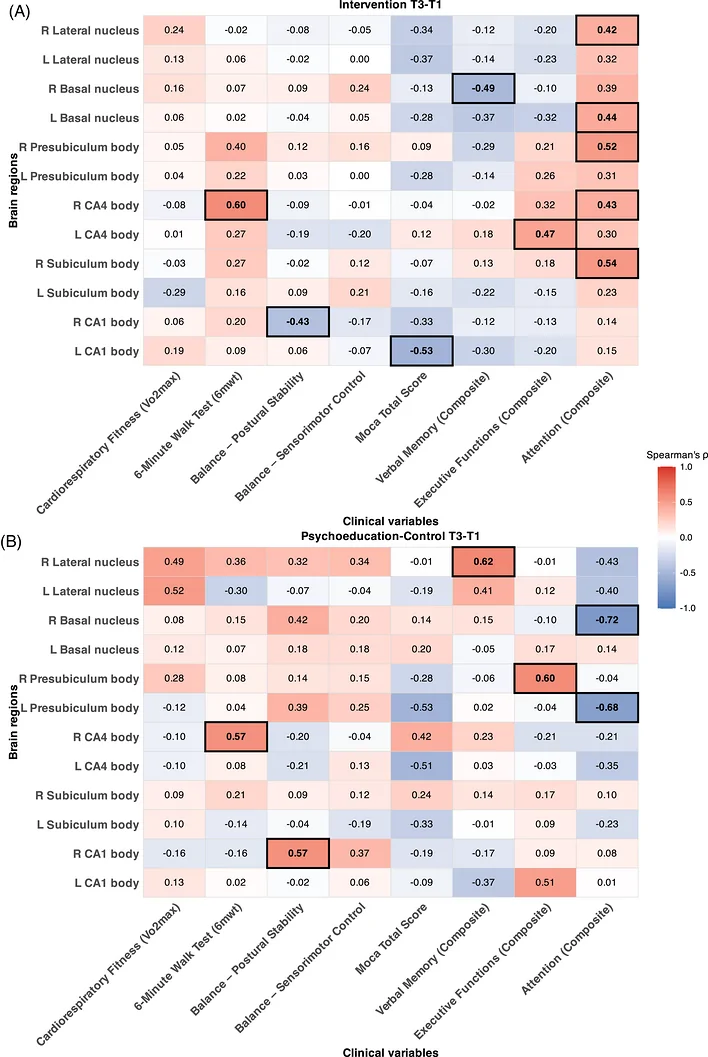
Correlation heatmaps illustrating associations between changes in clinical measures and longitudinal changes in functional connectivity within hippocampal subfield and amygdala nucleus networks across study groups for T_3_– T_1_ interval. (A) Intervention ΔT_3_– T_1_. (B) Psychoeducation control ΔT_3_– T_1_. Spearman's rank correlation was used to assess associations between change scores. Statistical significance was determined using false discovery rate (FDR) correction (*p* < 0.05), with bold values indicating significant correlations. Each cell in the heatmap represents a pairwise correlation between the change in a clinical marker and the corresponding change in a functional connectivity measure.

For the T_3_–T_1_ interval, changes in PSQI sleep measures were associated with changes in functional connectivity within hippocampal subfield and amygdala nucleus networks especially in the intervention group (Figure 4A,B). Associations were primarily negative and generally modest to moderate, with the most consistent patterns involving habitual sleep efficiency and global PSQI (Figure 4). The strongest negative correlation was found in the left basal nucleus, with significant negative correlation with sleep duration, habitual sleep efficiency, and global PSQI score (Figure 4A,B). For the same T_3_–T_1_ interval, changes in clinical measures were associated with changes in functional connectivity within hippocampus–amygdala networks. Significant associations involved mobility and balance measures and cognitive outcomes, with the highest associations of the attention composite score in the intervention group (Figure 5). Also, for brain volumetry, clinical variables correlated with volume change in hippocampal and amygdalar substructures, with the amygdalar substructures showing a negative correlation in the psychoeducation control group with the HADS, mainly driven by the depression subscore, between T_1_ and T_3_ (Figure S3). Correlation matrices of subfield volumes and ROI‐to‐ROI functional connectivity measures are additionally shown in Figure S4 to illustrate the degree of dependence between neighboring subfields and connections.

For the association between diary sleep data and volumetric and functional MRI results, the intervention group showed negative correlations between social jetlag hours and connectivity of the left subiculum, left and right CA1, left basal and paralaminar nucleus, right hippocampal fissure, right presubiculum, and right CA3 and CA4. For the second time part of the intervention, most associations were found in negative correlations between the weekend catchup hours and the left and right subiculum and presubiculum, CA1, left paralaminar nucleus, and the right hippocampal head (Supplementary Figure S5 and S6). For the intervention group, the correlation between hippocampal and amygdalar substructures and total sleep duration and Fitbit steps and MVPA was low. As a sensitivity check, analyses excluding participants without CSF biomarker confirmation or with documented obstructive sleep apnea showed unchanged results.

## 4 DISCUSSION

This multimodal longitudinal study examined how physical activity influenced hippocampal and amygdalar substructures and functional coupling in relation to sleep. Sleep was assessed using the PSQI at all time points, a sleep diary throughout the 6‐month intervention, and wearable devices at mid‐intervention. Three main findings emerged. First, the intervention arm showed physiological benefits, including increased VO_2_max and trend‐level balance/stability improvements, alongside relative preservation of hippocampal and amygdalar substructures during the intervention, with structural decline in both groups over 18 months. Second, hippocampus–amygdala functional connectivity increased during the intervention only in the intervention group, indicating intervention‐linked network reorganization. Third, volumetric and functional measures of hippocampal and amygdalar substructures were associated with PSQI, clinical, and functional outcomes, supporting the relevance of sleep patterns to MTL network reorganization and pointing to substantial adaptive changes in fitness, network organization, and behavior in the intervention group in spite of being more clinically affected.

At baseline, the psychoeducation control group showed better global cognition and a more favorable risk/pathology profile than the intervention group (1), indicating that the intervention arm started at a disadvantage. Nevertheless, during the 6‐month intervention, the intervention group showed physiological gains (VO_2_max) alongside comparatively attenuated hippocampal and amygdalar atrophy and increased hippocampus–amygdala connectivity. After intervention cessation, structural decline continued in both groups, consistent with ongoing neurodegenerative trajectories. Increased connectivity was inversely associated with PSQI scores, indicating that better subjective sleep quality was associated with increased connectivity. The basal nucleus of the amygdala emerged as a prominent region, having the strongest correlations with sleep duration, habitual sleep efficiency, and the global PSQI. The existing literature links disturbed or insufficient sleep to altered amygdala responsivity and circuit coupling. Insomnia studies report increased amygdala reactivity to insomnia‐related/negative cues, consistent with hyperarousal and PSQI domains such as sleep efficiency and perceived sleep quality.^67^, ^68^, ^69^ In our study, longitudinal hippocampus and amygdala measures were also associated with fitness, activity, and cognition, particularly attention and executive functions, linking network changes to physiological changes during the intervention.^67^ Amygdalar substructures were inversely associated with HADS depressive scores, especially in the psychoeducation control group between T_1_ and T_3_.

The maintenance of hippocampal‐amygdalar substructure volume during the intervention suggests short‐term stabilization, in line with work linking higher physical activity and cardiorespiratory fitness to preserved hippocampal volumes in older adults.^70^, ^71^, ^72^ Aerobic training increases anterior hippocampal volume and improves memory^73^ and higher fitness or habitual walking points to larger hippocampal subfields and slower atrophy.^41^, ^71^, ^72^ Resistance training benefits hippocampal subfield volumes in older adults at risk of MCI,^74^ with overall effects varying by baseline fitness, dose, and participant characteristics.^75^

This pattern of initial stabilization during intervention followed by post‐intervention decline likely reflects baseline neurodegenerative trajectories. Even intensive behavioral programs may slow but not reverse atrophy in vulnerable CA4/dentate gyrus and subiculum regions, where early tau and neuroinflammation accumulate,^76^, ^77^, ^78^ so neurodegeneration continues after the intervention ends.^79^ This parallels other neurodegenerative diseases such as Parkinson's disease, pointing to a needed long‐term engagement in activity for sustained positive effects.^80^ In this context, the relative amygdalar preservation is consistent with imaging studies showing earlier, steeper hippocampal versus amygdalar atrophy in prodromal or at‐risk states.^76^

In contrast to the predominantly degenerative structural pattern, functional connectivity within hippocampus–amygdala circuits showed exclusively increased connectivity in the intervention group. This pattern suggests more pronounced network‐level reorganization in the physically active group.^81^, ^82^ Taken together, the pattern of largely maintained brain volume during intervention accompanied by increased connectivity is more consistent with transient network reorganization or possible compensatory recruitment than with simple disconnection. Hyperconnectivity in MTL and default‐mode networks has been described in preclinical and early AD and may reflect upregulated synaptic and network activity amid accumulating pathology.^81^, ^82^, ^83^, ^84^ However, increased connectivity in AD is not uniformly beneficial and may reflect disease‐stage‐dependent hyperconnectivity or network inefficiency. These findings should therefore not be read as successful compensation but as intervention‐period changes in hippocampus–amygdala network coupling whose long‐term functional relevance remains uncertain, with the structured physical activity program associated with more extensive change than psychoeducation alone.

Hippocampus–amygdala measures were linked to changes in subjective sleep and in clinical and functional measures across intervals, consistent with prior work across the preclinical and prodromal AD spectrum.^20^, ^76^ Both volumes and connectivity strength correlated with multiple PSQI dimensions, including subjective sleep quality, sleep latency, daytime dysfunction, and global PSQI, with the strongest effects concentrated in specific subfields and nuclei rather than uniformly across the MTL. Sleep‐related connectivity associations were more frequent in the intervention group during the initial intervention interval, aligning with the broader pattern of more extensive connectivity change in the intervention group. Given the number of tested associations, individual correlations were interpreted cautiously, with emphasis on corrected results and consistent sleep‐related patterns. These findings converge with evidence that sleep efficiency and stability are related to hippocampal volume and memory in at‐risk older adults, that sleep fragmentation and shortened sleep are linked to fronto‐hippocampal hypometabolism and worse executive functioning, and that reduced sleep time predicts higher CSF tau in cognitively unimpaired elders. Large cohorts show that irregular or inefficient sleep is associated with accelerated cognitive decline and increased dementia risk, thereby representing an important target for intervention trials.^20^, ^85^, ^86^

Our data also underscore the importance of integrating sleep with 24‐h movement behaviors. Connectivity strength in specific subregions, including the parasubiculum, anterior amygdala area, and cortical amygdala nucleus, correlated with moderate and moderate to vigorous activity, consistent with studies linking walking, fitness, hippocampal plasticity, and preserved memory.^72^, ^73^ Combining multimodal neuroimaging with high‐density diary monitoring can capture real‐world sleep and activity signatures alongside imaging, supporting scalable behavioral biomarkers for ADRD risk stratification.^52^, ^86^, ^87^ MTL circuits appear sensitive not only to the amount of activity and sleep but also intensity, variability, and self‐regulation of health behaviors.

Interestingly, sleep‐related correlations were predominantly negative for both structural volumes and functional connectivity, whereas clinical correlations with fitness, balance, and cognition showed mixed directions depending on region and interval. Since lower PSQI reflects better sleep, the inverse correlation for functional connectivity reflects improved sleep being associated with increased connectivity and vice versa.

Together, these findings support the view that sleep and activity are closely linked with MTL health in older adults and should be treated as core components rather than ancillary variables in dementia prevention trials.^52^, ^86^, ^87^ The combination of hippocampus–amygdala subfield‐level volumetry and connectivity, CSF biomarkers, and 27‐week continuous data provides a detailed window into how lifestyle interventions interact with neurodegenerative processes.

Several limitations should be pointed out. Sample size limited statistical power, so uncorrected findings are exploratory; nonetheless, participants were well characterized, with most having CSF biomarker profiles, genetics, and strong clinical phenotyping often lacking in prior studies. Connectivity was interpreted at the atlas‐defined ROI level, as rs‐fMRI spatial resolution limits fine‐grained voxel‐level inference within small medial temporal subfields. Structural MRI was processed per time point using the same FastSurfer workflow rather than a longitudinal within‐subject template pipeline, possibly limiting sensitivity to subtle longitudinal changes. Baseline group differences in cognition, activity, biomarker, and cardiovascular profiles may have influenced longitudinal trajectories despite randomization, and the frequency and intensity of contact differed between arms, so non‐specific effects of supervision, motivation, and social interaction cannot be fully excluded. Although sleep was assessed continuously during the intervention phase (T_1_–T_3_) using daily sleep diaries, these scores are subject to variable adherence, missing entries, and potential reporter bias, which may limit their reliability and interpretability. The strain increase likely reflected intervention exposure, while the association with sleep duration should be interpreted cautiously as an individual‐level finding rather than evidence for a differential group effect on sleep trajectories. Also, the higher intake of sleep medication in the psychoeducation group at baseline might influence subjective sleep quality and sleep duration. Accordingly, inaccuracies related to self‐report cannot be ruled out entirely, even though self‐reported sleep measures, including the PSQI and sleep diaries, showed a high correlation with objective wearable‐derived sleep duration. Finally, although the 18‐month follow‐up is relatively long for an intensive behavioral protocol, it may still be insufficient to capture the full downstream impact of sleep and activity changes on structural decline.^76^ As mentioned in our methods section, we performed sensitivity analyses excluding the two participants without CSF biomarker data and the two participants with a documented diagnosis of obstructive sleep apnea, without changing the results. Nevertheless, a potential influence of biomarker‐related heterogeneity as well as undiagnosed sleep‐related breathing disorders or other sleep disorders cannot be fully excluded.

In summary, this study suggests that multimodal behavioral intervention in AD is accompanied by short‐term physiological benefits and hippocampus–amygdala network reorganization, and that MTL structure and connectivity are consistently associated with subjective sleep quality and 24‐h behavioral patterns. Structural atrophy in vulnerable hippocampal subfields progressed after cessation of the intervention, underlining the likely importance of sustained engagement. The convergence of subfield‐level volumetry, hippocampus–amygdala connectivity, clinical evaluation, and comprehensive real‐world sleep/activity monitoring highlights hippocampus–amygdala subfield measures as promising neuroimaging readouts for future interventions targeting sleep and circadian health in ADRD.

## AUTHOR CONTRIBUTIONS

R.D. and A.H. wrote the first draft of the manuscript. R.D. and A.H. interpreted the results of the data analysis. R.D. carried out programming and statistical analysis. A.H., R.D., and K.R. contributed to the conception and design of the study. A.H., A.S.C., K.R., S.D., R.D., and L.H. carried out planned data collection at the Aachen site. All authors reviewed and approved the manuscript.

## CONFLICT OF INTEREST STATEMENT

The authors report no competing interests. Author disclosures are available in the Supporting Information.

## CONSENT STATEMENT

All human subjects provided informed consent prior to participating in this study.

## Supporting information



Supporting Information

Supporting Information

## Data Availability

De‐identified study data and the analysis code used in this study are available from the corresponding author on reasonable request, subject to institutional and data protection regulations.
