# Supplementary material for: Longitudinal effects of physical activity on sleep in association with hippocampal‐amygdala subfield structure and functional connectivity in Alzheimer's disease
Source: Alzheimers Dement. 2026 Aug 25;22(8):e71651. doi: 10.1002/alz.71651 (PMC13504447; doi:10.1002/alz.71651)
Supplement: Supplementary file 2 [file ALZ-22-e71651-s001.docx]

**SUPPLEMENTARY DATA**

**Supplementary Text S1. MRIQC-based quality-control procedure**

MRIQC (v23.1.0) was used to generate image-quality metrics and visual reports for T1-weighted anatomical and resting-state BOLD scans.^1^ Quality control was performed in three steps: first, MRIQC outputs were checked for processing errors or failed report generation; second, anatomical and functional MRIQC reports were visually inspected; and third, BOLD scans were evaluated according to the predefined motion criteria described in the Methods section. For T1-weighted images, quality control focused on brain coverage, tissue contrast, signal quality, intensity non-uniformity, and visible artifacts. These checks were used to identify scans with gross artifacts or image-quality problems that could affect downstream segmentation or registration.

For BOLD scans, the main MRIQC outputs considered were those most relevant to resting-state data quality, particularly motion-related metrics and temporal stability measures. These included mean framewise displacement (fd_mean), the number and proportion of volumes with FD > 0.2 mm (fd_num and fd_perc), DVARS (dvars_nstd, dvars_std, and dvars_vstd) to identify abrupt volume-to-volume signal changes, temporal signal-to-noise ratio (tsnr) to assess temporal signal stability, global correlation (gcor) to assess shared global signal and noise, and entropy focus criterion (efc) to identify blurring or ghosting-related artifacts. Visual inspection focused on carpet plots, mean BOLD images, standard-deviation maps, ghosting, signal dropout, and visible EPI artifacts. Motion measures were prioritized because head motion is a major confound in resting-state functional connectivity analyses and because the functional exclusion criteria were defined a priori using framewise displacement.

Final exclusion from the functional analysis was based on predefined motion thresholds: participants were excluded if mean FD exceeded 0.30 mm, if more than 20% of volumes exceeded FD > 0.2 mm, or if any single frame exceeded FD > 5 mm. Other MRIQC outputs were used to support manual review and to flag scans for closer inspection but were not applied as separate independent exclusion thresholds.

**Supplementary Text S2. Detailed information of preprocessing pipeline for resting state fMRI**

**Anatomical data preprocessing**

A total of 1 T1-weighted (T1w) images were found within the input BIDS dataset. The T1w image was corrected for intensity non-uniformity (INU) with N4BiasFieldCorrection,^2^ distributed with ANTs 2.5.0 (RRID:SCR_004757),^3^ and used as T1w-reference throughout the workflow. The T1w-reference was then skull-stripped with a Nipype implementation of the antsBrainExtraction.sh workflow (from ANTs), using OASIS30ANTs as target template. Brain tissue segmentation of cerebrospinal fluid (CSF), white-matter (WM) and gray-matter (GM) was performed on the brain-extracted T1w using fast (FSL), RRID:SCR_002823, Zhang, Brady, and Smith 2001).^4^ Volume-based spatial normalization to one standard space (MNI152NLin2009cAsym) was performed through nonlinear registration with antsRegistration (ANTs 2.5.0), using brain-extracted versions of both T1w reference and the T1w template. The following template was were selected for spatial normalization and accessed with TemplateFlow (23.1.0,)^5^: ICBM 152 Nonlinear Asymmetrical template version 2009c [RRID:SCR_008796; TemplateFlow ID: MNI152NLin2009cAsym].^6^

**Preprocessing of B_0_ inhomogeneity mappings**

A total of 1 fieldmaps were found available within the input BIDS structure for this particular subject. A deformation field to correct for susceptibility distortions was estimated based on fMRIPrep’s fieldmap-less approach. The deformation field is that resulting from co-registering the EPI reference to the same-subject T1w-reference with its intensity inverted.^7,8^ Registration is performed with antsRegistration (ANTs 2.5.0), and the process regularized by constraining deformation to be nonzero only along the phase-encoding direction, and modulated with an average fieldmap template.^9^

**Functional data preprocessing**

For each of the 1 BOLD runs found per subject (across all tasks and sessions), the following preprocessing was performed. First, a reference volume was generated, using a custom methodology of fMRIPrep, for use in head motion correction. Head-motion parameters with respect to the BOLD reference (transformation matrices, and six corresponding rotation and translation parameters) are estimated before any spatiotemporal filtering using mcflirt (FSL)^10^ The estimated fieldmap was then aligned with rigid-registration to the target EPI (echo-planar imaging) reference run. The field coefficients were mapped on to the reference EPI using the transform. The BOLD reference was then co-registered to the T1w reference using mri_coreg (FreeSurfer) followed by flirt (FSL)^11^ with the boundary-based registration cost-function.^12^ Co-registration was configured with six degrees of freedom. Several confounding time-series were calculated based on the preprocessed BOLD: framewise displacement (FD), DVARS and three region-wise global signals. FD was computed using two formulations following Power (absolute sum of relative motions, relative root mean square displacement between affines).^10,13^ FD and DVARS are calculated for each functional run, both using their implementations in Nipype.^13^ The three global signals are extracted within the CSF, the WM, and the whole-brain masks. Additionally, a set of physiological regressors were extracted to allow for component-based noise correction (CompCor).^14^ Principal components are estimated after high-pass filtering the preprocessed BOLD time-series (using a discrete cosine filter with 128s cut-off) for the two CompCor variants: temporal (tCompCor) and anatomical (aCompCor). tCompCor components are then calculated from the top 2% variable voxels within the brain mask. For aCompCor, three probabilistic masks (CSF, WM and combined CSF+WM) are generated in anatomical space. The implementation differs from that of Behzadi et al. in that instead of eroding the masks by 2 pixels on BOLD space, a mask of pixels that likely contain a volume fraction of GM is subtracted from the aCompCor masks.^14^ This mask is obtained by thresholding the corresponding partial volume map at 0.05, and it ensures components are not extracted from voxels containing a minimal fraction of GM. Finally, these masks are resampled into BOLD space and binarized by thresholding at 0.99 (as in the original implementation). Components are also calculated separately within the WM and CSF masks. For each CompCor decomposition, the k components with the largest singular values are retained, such that the retained components’ time series are sufficient to explain 50 percent of variance across the nuisance mask (CSF, WM, combined, or temporal). The remaining components are dropped from consideration. The head-motion estimates calculated in the correction step were also placed within the corresponding confounds file. The confound time series derived from head motion estimates and global signals were expanded with the inclusion of temporal derivatives and quadratic terms for each.^15^ Frames that exceeded a threshold of 0.5 mm FD or 1.5 standardized DVARS were annotated as motion outliers. Additional nuisance timeseries are calculated by means of principal components analysis of the signal found within a thin band (crown) of voxels around the edge of the brain.^16^ All resamplings can be performed with a single interpolation step by composing all the pertinent transformations (i.e. head-motion transform matrices, susceptibility distortion correction when available, and co-registrations to anatomical and output spaces). Gridded (volumetric) resamplings were performed using nitransforms, configured with cubic B-spline interpolation. The presented description was generated automatically by fMRIPrep. which operates under the CC0 license and then modified by the authors of this work for clarity and accuracy.

**Supplementary Table 1. Baseline demographic characteristics and cerebrospinal fluid biomarkers of the structural and functional MRI groups**

| **Characteristic** | **Total** | **Intervention** | **Psychoeducation** | **p-value (FDR-corr.)** |
| --- | --- | --- | --- | --- |
| **Structural MRI** | n = 46 | n = 25 | n = 21 | - |
| Age, years | 70.7 ± 7.0 | 71.6 ± 5.8 | 68.6 ± 8.0 | 0.31 |
| Sex, female, n (%) | 18 (39.1) | 10 (40.0) | 8 (38.1) | 0.90 |
| Education, years | 13.3 ± 3.3 | 12.2 ± 3.7 | 14.6 ± 2.4 | 0.056 |
| Disease duration, years | 3.7 ± 1.9 | 3.8 ± 2.1 | 3.5 ± 1.7 | 0.66 |
| Clinical Dementia Rating (range) | 0.8 ± 0.4 (0-2) | 0.9 ± 0.3 (0.5-2) | 0.7 ± 0.4 (0-1.5) | 0.50 |
| Amyloid-β1-40 (pg/mL) | 10485.1 ± 4122.3 | 11083.6 ± 3571.5 | 9823.6 ± 4665.0 | 0.48 |
| Amyloid-β1-42 (pg/mL) | 410.7 ± 165.1 | 393.8 ± 162.9 | 428.4 ± 169.7 | 0.57 |
| Aβ1–42/Aβ1–40 ratio | 0.498 ± 0.580 | 0.360 ± 0.100 | 0.650 ± 0.820 | 0.27 |
| Phospho-Tau (pg/mL) | 85.6 ± 36.3 | 95.3 ± 35.9 | 76.0 ± 34.9 | 0.23 |
| Total Tau (pg/mL) | 598.7 ± 318.1 | 709.0 ± 317.8 | 488.5 ± 284.5 | 0.08 |
| **Functional MRI** | n = 42 | n = 23 | n = 19 | - |
| Age, years | 71.6 ± 5.8 | 71.2 ± 5.9 | 68.8 ± 7.9 | 0.60 |
| Sex, female, n (%) | 18 (43.9) | 10 (45.5) | 8 (42.1) | 0.99 |
| Education, years | 13.0 ± 3.4 | 12.0 ± 3.9 | 15.0 ± 2.2 | 0.12 |
| Disease duration, years | 4.0 ± 1.9 | 4.0 ± 2.1 | 4.0 ± 1.6 | 0.99 |
| Clinical Dementia Rating (range) | 0.8 ± 0.4 (0-2) | 0.8 ± 0.4 (0.5-2) | 0.7 ± 0.4 (0-1.5) | 0.329 |
| Amyloid-β1-40 (pg/mL) | 10424.7 ± 4137.3 | 10835.0 ± 3472.8 | 9942.1 ± 4871.7 | 0.733 |
| Amyloid-β1-42 (pg/mL) | 383.7 ± 137.3 | 376.0 ± 144.7 | 392.3 ± 132.3 | 0.820 |
| Aβ1–42/Aβ1–40 ratio | 0.5 ± 0.6 | 0.4 ± 0.1 | 0.6 ± 0.9 | 0.471 |
| Phospho-Tau (pg/mL) | 87.2 ± 36.6 | 96.9 ± 36.0 | 77.0 ± 35.2 | 0.327 |
| Total Tau (pg/mL) | 614.9 ± 322.7 | 723.0 ± 319.4 | 501.1 ± 292.5 | 0.175 |

*Mean values and standard deviations (SD) are presented for the imaging group. Composite scores represent z-scores. Reported p-values are FDR-corrected.

**Supplementary Table 2. Statistically significant within-group longitudinal changes in clinical outcomes for the intervention and psychoeducation groups**

| **Clinical Measure** | **Time Contrast** | **df** | **t-value** | **p-value** | **p-FDR** |
| --- | --- | --- | --- | --- | --- |
| **Intervention** | | | | | |
| Balance symmetry difference (mediolateral) | T4–T3 | 115.47 | 2.62 | 0.04 | 0.64 |
| HADS Depression subscale | T4–T3 | 107.75 | -2.68 | 0.04 | 0.64 |
| Montreal Cognitive Assessment | T4–T3 | 105.63 | -5.16 | 0.00 | 0.00 |
| Handgrip strength (right hand) | T4–T2 | 107.27 | -2.83 | 0.02 | 0.6 |
| Montreal Cognitive Assessment | T4–T2 | 105.63 | -4.75 | 0.00 | 0.00 |
| Postural stability index (anteroposterior) | T4–T2 | 105.72 | -3.11 | 0.01 | 0.37 |
| Sensorimotor balance index (mediolateral) | T4–T2 | 110.19 | -2.62 | 0.04 | 0.64 |
| Balance symmetry difference (mediolateral) | T4–T1 | 118.29 | 3.79 | 0.00 | 0.05 |
| Montreal Cognitive Assessment | T4–T1 | 106.2 | -6.19 | 0.00 | 0.00 |
| Postural stability index (anteroposterior) | T4–T1 | 107.35 | -3.26 | 0.00 | 0.3 |
| Sensorimotor balance index (mediolateral) | T4–T1 | 112.5 | -3.04 | 0.01 | 0.41 |
| Six-minute walk test distance | T4–T1 | 105.86 | -2.54 | 0.05 | 0.7 |
| HADS Depression subscale | T3–T1 | 106.37 | 4.22 | 0.00 | 0.02 |
| Hospital Anxiety and Depression Scale (total) | T3–T1 | 105.28 | 2.93 | 0.02 | 0.46 |
| ACT paq50 calories daily | T2–T1 | 83.74 | -2.5 | 0.03 | 0.64 |
| ACT paq50 met daily | T2–T1 | 83.79 | -2.48 | 0.04 | 0.64 |
| HADS Depression subscale | T2–T1 | 106.37 | 3.14 | 0.01 | 0.37 |
| **Psychoeducation** | | | | | |
| Hospital Anxiety and Depression Scale (total) | T2–T1 | 105.69 | 2.6 | 0.05 | 0.64 |
| Montreal Cognitive Assessment | T4–T3 | 106 | -4.02 | 0.00 | 0.03 |
| Montreal Cognitive Assessment | T4–T2 | 106 | -2.96 | 0.01 | 0.46 |
| Montreal Cognitive Assessment | T4–T1 | 106.82 | -3.98 | 0.00 | 0.03 |
| Handgrip strength (right hand) | T3–T1 | 107.36 | 2.74 | 0.03 | 0.63 |
| Pittsburgh Sleep Quality Index | T3–T1 | 83.7 | -2.42 | 0.04 | 0.64 |

**Supplementary Table 3. Statistically significant structural alterations in hippocampal and amygdala subfields in the intervention and psychoeducation groups across T1 to T4**

| **Region** | **Group** | **Term** | **p-value** | **p-FDR** | **Effect Size** | **Direction** |
| --- | --- | --- | --- | --- | --- | --- |
| **Hippocampus Subfield Regions** | | | | | | |
| Lh Fimbria | Intervention | Time | 0.046945 | 0.175261 | -2.11288 | Decreasing |
| Rh Subiculum Head | Intervention | Time | 0.003059 | 0.027412 | -3.35191 | Decreasing |
| Rh Presubiculum Head | Intervention | Time | 0.000144 | 0.004249 | -4.44052 | Decreasing |
| Rh Presubiculum Body | Intervention | Time | 0.012892 | 0.072195 | -2.58564 | Decreasing |
| Rh Ca3 Body | Intervention | Time | 0.039739 | 0.156626 | -2.16431 | Decreasing |
| Rh Gc Ml Dg Body | Intervention | Time | 0.002342 | 0.022812 | -3.373 | Decreasing |
| Rh Ca4 Body | Intervention | Time | 0.003775 | 0.030197 | -3.03836 | Decreasing |
| Rh Fimbria | Intervention | Time | 0.007725 | 0.048214 | -2.80198 | Decreasing |
| Rh Whole Hippocampal Body | Intervention | Time | 0.028157 | 0.119685 | -2.3406 | Decreasing |
| **Amygdala Subfield Regions** | | | | | | |
| Right Corticoamygdaloid Transitio | Intervention | Time | 0.0263 | 0.114 | -2.353 | Decreasing |

***** Note. Only uncorrected effects (p < 0.05) in hippocampal and amygdala subfields are shown. All uncorrected significant effects occurred in the intervention group; no region in the psychoeducation group reached the uncorrected significance threshold.

**Supplementary Table 4. Statistically significant structural alterations in hippocampal and amygdala subfields across follow-up comparisons in the intervention and psychoeducation groups**

| **Region** | **Group** | **Term** | **p-value** | **p-FDR** | **Effect Size** | **Direction** |
| --- | --- | --- | --- | --- | --- | --- |
| **Hippocampus Subfield Regions** | | | | | | |
| Left Subiculum Body | Psychoeducation | T3-T1 | 0.031717 | 0.328395 | -2.20292 | Decreasing |
| Left Hippocampal Fissure | Psychoeducation | T3-T1 | 0.031396 | 0.328395 | -2.20685 | Decreasing |
| Left Ca1 Head | Psychoeducation | T3-T1 | 0.042295 | 0.377837 | -2.07797 | Decreasing |
| Left Gc Ml Dg Head | Psychoeducation | T3-T1 | 0.020642 | 0.324231 | -2.38124 | Decreasing |
| Left Gc Ml Dg Body | Psychoeducation | T3-T1 | 0.014025 | 0.308038 | -2.53542 | Decreasing |
| Left Ca4 Head | Psychoeducation | T3-T1 | 0.029522 | 0.328395 | -2.23291 | Decreasing |
| Left Ca4 Body | Psychoeducation | T3-T1 | 0.015161 | 0.308038 | -2.50496 | Decreasing |
| Left Fimbria | Intervention | T4-T1 | 0.023471 | 0.324231 | -2.32179 | Decreasing |
| Left Fimbria | Psychoeducation | T4-T1 | 0.025406 | 0.324231 | -2.29191 | Decreasing |
| Left Ca3 Head | Psychoeducation | T3-T1 | 0.024424 | 0.324231 | -2.3122 | Decreasing |
| Left Whole Hippocampus | Psychoeducation | T3-T1 | 0.022526 | 0.324231 | -2.34601 | Decreasing |
| Left Whole Hippocampal Body | Psychoeducation | T3-T1 | 0.016092 | 0.308038 | -2.48148 | Decreasing |
| Left Whole Hippocampal Head | Psychoeducation | T3-T1 | 0.029313 | 0.328395 | -2.23639 | Decreasing |
| Right Hippocampal Tail | Psychoeducation | T4-T1 | 0.033203 | 0.329573 | -2.1766 | Decreasing |
| Right Subiculum Body | Psychoeducation | T3-T1 | 0.019666 | 0.324231 | -2.4024 | Decreasing |
| Right Subiculum Head | Intervention | T4-T1 | 0.001241 | 0.308038 | -3.3755 | Decreasing |
| Right Subiculum Head | Intervention | T4-T3 | 0.007919 | 0.308038 | -2.74449 | Decreasing |
| Right Presubiculum Head | Intervention | T4-T1 | 0.003008 | 0.308038 | -3.08129 | Decreasing |
| Right Presubiculum Head | Intervention | T4-T3 | 0.009325 | 0.308038 | -2.68369 | Decreasing |
| Right Presubiculum Head | Psychoeducation | T3-T1 | 0.048577 | 0.382927 | -2.01683 | Decreasing |
| Right Presubiculum Body | Psychoeducation | T4-T1 | 0.031859 | 0.328395 | -2.19584 | Decreasing |
| Right Molecular Layer Hp Body | Psychoeducation | T4-T3 | 0.038439 | 0.355227 | 2.119042 | Increasing |
| Right Ca3 Body | Intervention | T4-T1 | 0.013221 | 0.308038 | -2.54571 | Decreasing |
| Right Ca3 Body | Intervention | T3-T1 | 0.037904 | 0.355227 | -2.12762 | Decreasing |
| Right Gc Ml Dg Body | Intervention | T4-T1 | 0.005064 | 0.308038 | -2.89908 | Decreasing |
| Right Ca4 Body | Intervention | T4-T1 | 0.005559 | 0.308038 | -2.868 | Decreasing |
| Right Fimbria | Intervention | T4-T1 | 0.010901 | 0.308038 | -2.62409 | Decreasing |
| Right Ca3 Head | Psychoeducation | T3-T1 | 0.047386 | 0.382927 | -2.02753 | Decreasing |
| Right Whole Hippocampus | Psychoeducation | T3-T1 | 0.020916 | 0.324231 | -2.37739 | Decreasing |
| Right Whole Hippocampal Body | Intervention | T4-T1 | 0.046198 | 0.382927 | -2.03018 | Decreasing |
| Right Whole Hippocampal Body | Psychoeducation | T3-T1 | 0.010293 | 0.308038 | -2.65653 | Decreasing |
| Right Whole Hippocampal Head | Psychoeducation | T3-T1 | 0.049454 | 0.382927 | -2.00874 | Decreasing |
| **Amygdala Subfield Regions** | | | | | | |
| Left Lateral Nucleus | Psychoeducation | T3-T1 | 0.033133 | 0.356217 | -2.18369 | Decreasing |
| Left Basal Nucleus | Psychoeducation | T3-T1 | 0.03401 | 0.356217 | -2.17314 | Decreasing |
| Left Accessory Basal Nucleus | Psychoeducation | T3-T1 | 0.036819 | 0.356217 | -2.13921 | Decreasing |
| Left Corticoamygdaloid Transitio | Psychoeducation | T3-T1 | 0.036281 | 0.356217 | -2.14517 | Decreasing |
| Left Paralaminar Nucleus | Psychoeducation | T3-T1 | 0.039169 | 0.356217 | -2.11127 | Decreasing |
| Left Whole Amygdala | Psychoeducation | T3-T1 | 0.028909 | 0.356217 | -2.24225 | Decreasing |
| Right Lateral Nucleus | Psychoeducation | T3-T1 | 0.025857 | 0.356217 | -2.28978 | Decreasing |
| Right Accessory Basal Nucleus | Psychoeducation | T4-T1 | 0.040218 | 0.356217 | -2.09525 | Decreasing |
| Right Accessory Basal Nucleus | Psychoeducation | T3-T1 | 0.017231 | 0.356217 | -2.45566 | Decreasing |
| Right Anterior Amygdaloid Area Aaa | Psychoeducation | T4-T1 | 0.045198 | 0.367103 | -2.04292 | Decreasing |
| Right Central Nucleus | Psychoeducation | T3-T1 | 0.024263 | 0.356217 | -2.31562 | Decreasing |
| Right Medial Nucleus | Psychoeducation | T3-T1 | 0.037072 | 0.356217 | -2.13559 | Decreasing |
| Right Whole Amygdala | Psychoeducation | T3-T1 | 0.023531 | 0.356217 | -2.32904 | Decreasing |

***** Note. Only uncorrected effects (p < 0.05) in hippocampal and amygdala subfields are shown. No findings survived multiple-comparisons correction.

**Supplementary Table 5. Comparison of statistically significant structural brain changes between intervention and psychoeducation groups**

| **Region** | **Time** | **Group** | **df** | **p-value** | **p-FDR** | **Effect Size** | **Direction** |
| --- | --- | --- | --- | --- | --- | --- | --- |
| **Hippocampus Subfield Regions** | | | | | | | |
| Right Subiculum Head | T4 | Intervention - Psychoeducation | 57.72 | 0.030 | 0.86 | -2.21 | Decreasing |
| Right Fimbria | T1 | Intervention - Psychoeducation | 47.83 | 0.038 | 0.86 | -2.13 | Decreasing |
| Right Fimbria | T3 | Intervention - Psychoeducation | 49.10 | 0.037 | 0.86 | -2.13 | Decreasing |
| Right Fimbria | T4 | Intervention - Psychoeducation | 68.00 | 0.035 | 0.86 | -2.14 | Decreasing |
| **Amygdala Subfield Regions** | | | | | | | |
| Left Cortical Nucleus | T1 | Intervention - Psychoeducation | 52.67 | 0.03 | 0.44 | -2.17 | Decreasing |

***** Note. Only uncorrected effects (p < 0.05) in hippocampal and amygdala subfields are shown. No findings survived multiple-comparisons correction.

**Supplementary Table 6. Descriptive statistics for analyzed hippocampal and amygdala subfield volumes**

| **Subfield** | **Mean ± SD** | **Min volume** | **Max volume** | **Range** |
| --- | --- | --- | --- | --- |
| **Hippocampus** | | | | |
| Left Hippocampal Tail | 472.60 ± 95.12 | 242.34 | 653.23 | 242.34–653.23 |
| Left Subiculum Body | 224.23 ± 49.10 | 116.7 | 346.97 | 116.70–346.97 |
| Left CA1 Body | 118.49 ± 27.41 | 50.94 | 170.98 | 50.94–170.98 |
| Left Subiculum Head | 163.86 ± 35.71 | 92.57 | 231.7 | 92.57–231.70 |
| Left Hippocampal Fissure | 180.97 ± 29.84 | 122.22 | 234.33 | 122.22–234.33 |
| Left Presubiculum Head | 118.73 ± 27.03 | 58.85 | 170.29 | 58.85–170.29 |
| Left CA1 Head | 482.34 ± 97.57 | 291.48 | 665.01 | 291.48–665.01 |
| Left Presubiculum Body | 158.47 ± 33.09 | 102.66 | 225.18 | 102.66–225.18 |
| Left Parasubiculum | 62.58 ± 20.65 | 22.02 | 118.11 | 22.02–118.11 |
| Left Molecular Layer HP Head | 169.98 ± 41.65 | 91.69 | 264.32 | 91.69–264.32 |
| Left Molecular Layer HP Body | 117.60 ± 31.81 | 66.53 | 222.93 | 66.53–222.93 |
| Left GC-ML-DG Head | 139.24 ± 30.33 | 85.68 | 199.42 | 85.68–199.42 |
| Left CA3 Body | 80.36 ± 17.75 | 47.56 | 121.22 | 47.56–121.22 |
| Left GC-ML-DG Body | 118.16 ± 19.60 | 77.54 | 156.89 | 77.54–156.89 |
| Left CA4 Head | 119.95 ± 25.68 | 77.79 | 172.28 | 77.79–172.28 |
| Left CA4 Body | 109.01 ± 18.28 | 74.81 | 145.27 | 74.81–145.27 |
| Left Fimbria | 37.49 ± 19.89 | 2.82 | 75.18 | 2.82–75.18 |
| Left CA3 Head | 106.37 ± 20.95 | 70.14 | 154.15 | 70.14–154.15 |
| Left HATA | 46.16 ± 10.87 | 24.44 | 67.5 | 24.44–67.50 |
| Left Whole Hippocampus | 2845.64 ± 505.61 | 1815.22 | 3734.7 | 1815.22–3734.70 |
| Left Whole Hippocampal Body | 963.83 ± 169.92 | 607.98 | 1284.02 | 607.98–1284.02 |
| Left Whole Hippocampal Head | 1409.21 ± 270.46 | 869.27 | 1912.47 | 869.27–1912.47 |
| Right Hippocampal Tail | 508.61 ± 86.62 | 321.09 | 665.36 | 321.09–665.36 |
| Right Subiculum Body | 223.38 ± 46.53 | 127.14 | 341.88 | 127.14–341.88 |
| Right CA1 Body | 127.97 ± 30.63 | 54.8 | 211.85 | 54.80–211.85 |
| Right Subiculum Head | 157.78 ± 29.76 | 97.7 | 214.94 | 97.70–214.94 |
| Right Hippocampal Fissure | 187.64 ± 24.44 | 130.87 | 224.5 | 130.87–224.50 |
| Right Presubiculum Head | 112.16 ± 22.47 | 60.41 | 149.03 | 60.41–149.03 |
| Right CA1 Head | 490.78 ± 95.79 | 270.66 | 702.71 | 270.66–702.71 |
| Right Presubiculum Body | 146.71 ± 27.53 | 89.08 | 212.25 | 89.08–212.25 |
| Right Parasubiculum | 55.33 ± 14.48 | 21.4 | 92.63 | 21.40–92.63 |
| Right Molecular Layer HP Head | 163.72 ± 33.62 | 92.16 | 228.6 | 92.16–228.60 |
| Right Molecular Layer HP Body | 110.22 ± 24.44 | 67.76 | 190.31 | 67.76–190.31 |
| Right GC-ML-DG Head | 147.21 ± 29.62 | 75.12 | 223.01 | 75.12–223.01 |
| Right CA3 Body | 91.77 ± 21.08 | 49.62 | 152.45 | 49.62–152.45 |
| Right GC-ML-DG Body | 122.00 ± 22.25 | 79.66 | 189.44 | 79.66–189.44 |
| Right CA4 Head | 128.73 ± 25.02 | 70.39 | 190.19 | 70.39–190.19 |
| Right CA4 Body | 113.45 ± 19.71 | 79.32 | 168.38 | 79.32–168.38 |
| Right Fimbria | 33.43 ± 17.84 | 3.31 | 74.36 | 3.31–74.36 |
| Right CA3 Head | 115.66 ± 22.83 | 67.64 | 180.92 | 67.64–180.92 |
| Right HATA | 47.09 ± 11.31 | 24.19 | 71.71 | 24.19–71.71 |
| Right Whole Hippocampus | 2896.00 ± 477.60 | 1743.25 | 3886.25 | 1743.25–3886.25 |
| Right Whole Hippocampal Body | 968.94 ± 171.79 | 606.83 | 1429.28 | 606.83–1429.28 |
| Right Whole Hippocampal Head | 1418.44 ± 248.95 | 815.33 | 1849.96 | 815.33–1849.96 |
| **Amygdala** | | | | |
| Left Lateral Nucleus | 545.97 ± 101.41 | 361.34 | 780.56 | 361.34–780.56 |
| Left Basal Nucleus | 364.68 ± 76.83 | 223.7 | 540.56 | 223.70–540.56 |
| Left Accessory Basal Nucleus | 211.49 ± 53.13 | 131.16 | 330.65 | 131.16–330.65 |
| Left Anterior Amygdaloid Area | 42.19 ± 8.44 | 27.24 | 63.55 | 27.24–63.55 |
| Left Central Nucleus | 40.25 ± 14.15 | 21.1 | 73.24 | 21.10–73.24 |
| Left Medial Nucleus | 22.39 ± 7.57 | 9.16 | 38.34 | 9.16–38.34 |
| Left Cortical Nucleus | 22.80 ± 7.17 | 9.33 | 37.97 | 9.33–37.97 |
| Left Corticoamygdaloid Transition | 148.43 ± 30.39 | 91.26 | 208.61 | 91.26–208.61 |
| Left Paralaminar Nucleus | 43.93 ± 7.69 | 25.91 | 63.12 | 25.91–63.12 |
| Left Whole Amygdala | 1442.12 ± 291.99 | 924.6 | 2089.85 | 924.60–2089.85 |
| Right Lateral Nucleus | 556.22 ± 103.34 | 339.34 | 761.86 | 339.34–761.86 |
| Right Basal Nucleus | 371.83 ± 70.30 | 210.54 | 490.67 | 210.54–490.67 |
| Right Accessory Basal Nucleus | 223.97 ± 50.59 | 116.23 | 323.16 | 116.23–323.16 |
| Right Anterior Amygdaloid Area | 44.95 ± 8.69 | 28.43 | 61.73 | 28.43–61.73 |
| Right Central Nucleus | 42.54 ± 11.77 | 22.47 | 72.16 | 22.47–72.16 |
| Right Medial Nucleus | 25.52 ± 6.65 | 13.85 | 40.59 | 13.85–40.59 |
| Right Cortical Nucleus | 25.68 ± 5.96 | 12.83 | 40.03 | 12.83–40.03 |
| Right Corticoamygdaloid Transition | 150.28 ± 28.61 | 82.55 | 211.35 | 82.55–211.35 |
| Right Paralaminar Nucleus | 43.82 ± 7.16 | 26.79 | 55.8 | 26.79–55.80 |
| Right Whole Amygdala | 1484.82 ± 280.24 | 855.38 | 2047.01 | 855.38–2047.01 |

*Values are shown for baseline measurements from participants with available hippocampal and amygdala subfield segmentation outputs (41 of 46 baseline participants). Volumes are reported in mm³. Mean ± SD, minimum, maximum, and range are reported for each analysed subfield. Missingness was 0% for all hippocampal and amygdala subfield measurements included in this table.

**Supplementary Table 7. Statistically significant longitudinal ROI-to-ROI functional connectivity changes from T1 to T3 in the intervention and psychoeducation groups**

| **Brain Region** | **Statistic** | **p-value** | **p-FDR** | **p-Bonferroni** |
| --- | --- | --- | --- | --- |
| **Intervention ROI-ROI Connectivity** | | | | |
| Left lateral nucleus - Right presubiculum head | T(35) = 4.80 | 0.00003 | 0.049043 | 0.00066 |
| Right gc ml dg body - Left basal nucleus | T(35) = 3.78 | 0.000581 | 0.479981 | 0.012782 |
| Left ca4 body - Right presubiculum head | T(35) = 3.52 | 0.001213 | 0.59023 | 0.026686 |
| Left molecular layer hp body - Right hippocampal fissure | T(35) = 3.37 | 0.001861 | 0.59023 | 0.040942 |
| Left lateral nucleus - Right hippocampal fissure | T(35) = 3.28 | 0.002339 | 0.59023 | 0.051458 |
| Left gc ml dg body - Right presubiculum head | T(35) = 3.26 | 0.002465 | 0.59023 | 0.05423 |
| Right molecular layer hp body - Left basal nucleus | T(35) = 3.26 | 0.002499 | 0.59023 | 0.054978 |
| Left lateral nucleus - Right molecular layer hp head | T(35) = 3.08 | 0.003995 | 0.634042 | 0.08789 |
| Right gc ml dg body - Left lateral nucleus | T(35) = 3.06 | 0.004245 | 0.634042 | 0.09339 |
| Right molecular layer hp body - Left lateral nucleus | T(35) = 3.05 | 0.004326 | 0.634042 | 0.095172 |
| Left molecular layer hp body - Right presubiculum head | T(35) = 3.04 | 0.004497 | 0.634042 | 0.098934 |
| Left ca4 body - Right hippocampal fissure | T(35) = 3.03 | 0.004603 | 0.634042 | 0.101266 |
| Right ca4 body - Left basal nucleus | T(35) = 2.97 | 0.00535 | 0.680216 | 0.1177 |
| Right subiculum body - Left ca1 head | T(35) = 2.93 | 0.005858 | 0.69163 | 0.128876 |
| Left ca3 head - Left subiculum head | T(35) = -2.87 | 0.006869 | 0.729014 | 0.151118 |
| Left gc ml dg body - Right hippocampal fissure | T(35) = 2.85 | 0.007364 | 0.729014 | 0.162008 |
| Left molecular layer hp body - Left basal nucleus | T(35) = 2.84 | 0.007497 | 0.729014 | 0.164934 |
| Right subiculum body - Left lateral nucleus | T(35) = 2.78 | 0.008789 | 0.733814 | 0.193358 |
| Left parasubiculum - Left presubiculum head | T(35) = 2.76 | 0.00922 | 0.733814 | 0.20284 |
| Right subiculum body - Left basal nucleus | T(35) = 2.74 | 0.009545 | 0.733814 | 0.20999 |
| Right fimbria - Left basal nucleus | T(35) = 2.73 | 0.009811 | 0.733814 | 0.215842 |
| Left ca3 body - Right presubiculum head | T(35) = 2.72 | 0.009973 | 0.733814 | 0.219406 |
| **Psychoeducation ROI-ROI Connectivity** | | | |  |
| Right gc ml dg body - Right anterior amygdaloid area aaa | T(35) = 3.66 | 0.000814 | 0.509164 | 0.008954 |
| Right subiculum body - Right anterior amygdaloid area aaa | T(35) = 3.59 | 0.001014 | 0.509164 | 0.011154 |
| Left ca4 body - Right basal nucleus | T(35) = 3.58 | 0.001042 | 0.509164 | 0.011462 |
| Right hippocampal tail - Right ca3 head | T(35) = -3.49 | 0.001324 | 0.509164 | 0.014564 |
| Right ca4 body - Right anterior amygdaloid area aaa | T(35) = 3.44 | 0.00154 | 0.509164 | 0.01694 |
| Right hata - Right paralaminar nucleus | T(35) = 3.31 | 0.002172 | 0.598375 | 0.023892 |
| Left ca4 body - Right lateral nucleus | T(35) = 3.25 | 0.002534 | 0.598375 | 0.027874 |
| Left gc ml dg body - Right basal nucleus | T(35) = 3.11 | 0.003688 | 0.762109 | 0.040568 |
| Right anterior amygdaloid area aaa - Right alveus | T(35) = 2.85 | 0.007223 | 0.99989 | 0.079453 |
| Left gc ml dg body - Right lateral nucleus | T(35) = 2.75 | 0.009255 | 0.99989 | 0.101805 |
| Right hippocampal tail - Right presubiculum head | T(35) = -2.74 | 0.009578 | 0.99989 | 0.105358 |

* Note. ROI-to-ROI connectivity results at ROI level; thresholds: p-unc < 0.01, p-FDR < 0.05, p-Bonferroni < 0.05. Positive t-values indicate increased functional connectivity, whereas negative t-values indicate decreased functional connectivity.

**Supplementary Table 8. ROI-to-ROI functional connectivity differences between intervention and psychoeducation groups at each time point**

| **Brain Region** | **Time** | **Group** | **Statistic** | **p-value** | **p-FDR** | **p-Bonferroni** |
| --- | --- | --- | --- | --- | --- | --- |
| Left hata - Left corticoamygdaloid transitio | T1 | Intervention - Psychoeducation | T(35) = 3.17 | 0.003123 | 0.995235 | 0.021861 |
| Right gc ml dg body - Right subiculum head | T1 | Intervention - Psychoeducation | T(35) = 3.11 | 0.003668 | 0.995235 | 0.025676 |
| Right parasubiculum - Right hippocampal fissure | T1 | Intervention - Psychoeducation | T(35) = 3.03 | 0.004521 | 0.995235 | 0.031647 |
| Right medial nucleus - Right cortical nucleus | T1 | Intervention - Psychoeducation | T(35) = 3.01 | 0.004769 | 0.995235 | 0.033383 |
| Right ca4 body - Right subiculum head | T1 | Intervention - Psychoeducation | T(35) = 2.86 | 0.007115 | 0.995235 | 0.049805 |
| Right molecular layer hp body - Right subiculum head | T1 | Intervention - Psychoeducation | T(35) = 2.80 | 0.008313 | 0.995235 | 0.058191 |
| Left lateral nucleus - Left accessory basal nucleus | T1 | Intervention - Psychoeducation | T(35) = -2.78 | 0.008588 | 0.995235 | 0.060116 |
| Left ca3 body - Left fimbria | T3 | Intervention - Psychoeducation | T(35) = 3.69 | 0.000754 | 0.915813 | 0.004524 |
| Right subiculum body - Left medial nucleus | T3 | Intervention - Psychoeducation | T(35) = 3.00 | 0.005012 | 0.915813 | 0.030072 |
| Right anterior amygdaloid area aaa - Right alveus | T3 | Intervention - Psychoeducation | T(35) = -2.89 | 0.006569 | 0.915813 | 0.039414 |
| Left molecular layer hp body - Left fimbria | T3 | Intervention - Psychoeducation | T(35) = 2.88 | 0.006774 | 0.915813 | 0.040644 |
| Right hata - Right paralaminar nucleus | T3 | Intervention - Psychoeducation | T(35) = -2.82 | 0.007903 | 0.915813 | 0.047418 |
| Left presubiculum body - Left basal nucleus | T3 | Intervention - Psychoeducation | T(35) = 2.77 | 0.008986 | 0.915813 | 0.053916 |

* Note. ROI-to-ROI connectivity results at ROI level; thresholds: p-unc < 0.01, p-FDR < 0.05, p-Bonferroni < 0.05. Positive t-values indicate increased functional connectivity, whereas negative t-values indicate decreased functional connectivity.

Supplementary Figure 1. Spearman’s correlations between Fitbit-derived total sleep duration and selected self-reported sleep-duration measures in the intervention group. Bars show Spearman’s correlation coefficients (r); p-values are FDR-adjusted, with statistical significance set at p < 0.05.

Supplementary Figure 2. Correlation heatmaps illustrating the associations between changes in PSQI sleep measures and longitudinal changes in hippocampal subfield and amygdala nuclei volumes across study groups and time intervals. Panels represent: (A) intervention ΔT4–T3, (B) psychoeducation ΔT4–T3. Spearman’s rank correlation was used to assess associations between change scores. Statistical significance was determined using false discovery rate (FDR) correction (p < 0.05), with bold values indicating significant correlations. Each cell in the heatmap represents a pairwise correlation between the change in a clinical marker and the corresponding change in a hippocampal subfield or amygdala nucleus volume.

Supplementary Figure 3. Correlation heatmaps illustrating the associations between changes in clinical measures and longitudinal changes in hippocampal subfield and amygdala nuclei volumes across study groups and time intervals. Panels represent: (A) intervention ΔT3–T1, (B) intervention ΔT4–T3, (C) intervention ΔT4–T1, (D) psychoeducation ΔT3–T1, (E) psychoeducation ΔT4–T1, and (F) psychoeducation ΔT4–T3. Spearman’s rank correlation was used to assess associations between change scores. Statistical significance was determined using false discovery rate (FDR) correction (p < 0.05), with bold values indicating significant correlations. Each cell in the heatmap represents a pairwise correlation between the change in a clinical marker and the corresponding change in a hippocampal subfield or amygdala nucleus volume.

Supplementary Figure 4. Correlation heatmaps illustrating associations between longitudinal changes in structural brain regions and functional brain regions across study groups. Panels represent: (A) intervention ΔT3–T1 and (B) psychoeducation-control ΔT3–T1. Each cell shows the Spearman’s correlation coefficient between longitudinal structural change and longitudinal functional change. Statistical significance was assessed using Spearman’s rank correlation with false discovery rate (FDR) correction (p < 0.05), with bold values and black-bordered cells indicating significant correlations after correction.

Supplementary Figure 5. Correlation heatmaps showing associations between structural brain-region volumes and longitudinal sleep parameters derived from 27-week sleep diaries. Panels represent correlations for (A) Intervention T1 (months 1–3), (B) Psychoeducation control T1 (months 1–3), (C) Intervention T3 (months 4–6), and (D) Psychoeducation control T3 (months 4–6). Each cell displays the Spearman's rank correlation coefficient (ρ) between structural brain-region volumes (rows) and longitudinal sleep parameters derived from 27-week sleep diaries (columns). Correlations were calculated separately within each study group and assessment period. Bold values enclosed by black-bordered cells indicate statistically significant correlations after false discovery rate (FDR) correction (adjusted p < 0.05). Warmer colors represent positive correlations, cooler colors represent negative correlations, and white indicates no association.

Supplementary Figure 6. Correlation heatmaps showing associations between functional brain connectivity measures and longitudinal sleep parameters derived from 27-week sleep diaries. Panels represent correlations for (A) Intervention T1 (months 1–3), (B) Psychoeducation control T1 (months 1–3), (C) Intervention T3 (months 4–6), and (D) Psychoeducation control T3 (months 4–6). Each cell displays the Spearman's rank correlation coefficient (ρ) between functional brain connectivity measures (rows) and sleep parameters (columns). Sleep parameters include mean sleep duration, long nights, short nights, sleep data completeness, sleep duration variability (hours and coefficient of variation), and social jetlag, averaged over the corresponding three-month period. Correlations were calculated separately within each study group and assessment period. Bold values enclosed by black borders indicate statistically significant correlations after false discovery rate (FDR) correction (adjusted p < 0.05). Warmer colors represent positive correlations, cooler colors represent negative correlations, and white indicates no association.

**References**

1.	Esteban O, Birman D, Schaer M, Koyejo OO, Poldrack RA, Gorgolewski KJ. MRIQC: Advancing the automatic prediction of image quality in MRI from unseen sites. *PLoS One*. 2017;12(9):e0184661. doi:10.1371/journal.pone.0184661

2.	Tustison NJ, Avants BB, Cook PA, et al. N4ITK: improved N3 bias correction. *IEEE Trans Med Imaging*. 2010;29(6):1310-1320. doi:10.1109/TMI.2010.2046908

3.	Avants BB, Epstein CL, Grossman M, Gee JC. Symmetric diffeomorphic image registration with cross-correlation: Evaluating automated labeling of elderly and neurodegenerative brain. *Medical Image Analysis*. 2008;12(1):26-41. doi:10.1016/j.media.2007.06.004

4.	Zhang Y, Brady M, Smith S. Segmentation of brain MR images through a hidden Markov random field model and the expectation-maximization algorithm. *IEEE Transactions on Medical Imaging*. 2001;20(1):45-57. doi:10.1109/42.906424

5.	Ciric R, Thompson WH, Lorenz R, et al. TemplateFlow: FAIR-Sharing of Multi-Scale, Multi-Species Brain Models. *Nature Methods*. 2022;19:1568-1571. doi:10.1038/s41592-022-01681-2

6.	Fonov V, Evans A, McKinstry R, Almli C, Collins D. Unbiased nonlinear average age-appropriate brain templates from birth to adulthood. *NeuroImage*. 2009;47:S102. doi:10.1016/S1053-8119(09)70884-5

7.	Wang M, Beal DJ, Chan D, Newman DA, Vancouver JB, Vandenberg RJ. Longitudinal Research: A Panel Discussion on Conceptual Issues, Research Design, and Statistical Techniques. *Work, Aging and Retirement*. 2017;3(1):1-24. doi:10.1093/workar/waw033

8.	Huntenburg JM. *Evaluating Nonlinear Coregistration of BOLD EPI and T1w Images*. Master’s Thesis. Freie Universität Berlin; 2014. https://hdl.handle.net/11858/00-001M-0000-002B-1CB5-A

9.	Treiber JM, White NS, Steed TC, et al. Characterization and Correction of Geometric Distortions in 814 Diffusion Weighted Images. *PLOS ONE*. 2016;11(3):e0152472. doi:10.1371/journal.pone.0152472

10.	Jenkinson M, Bannister P, Brady M, Smith S. Improved optimization for the robust and accurate linear registration and motion correction of brain images. *Neuroimage*. 2002;17(2):825-841. doi:10.1016/s1053-8119(02)91132-8

11.	Jenkinson M, Smith S. A Global Optimisation Method for Robust Affine Registration of Brain Images. *Medical Image Analysis*. 2001;5(2):143-156. doi:10.1016/S1361-8415(01)00036-6

12.	Greve DN, Fischl B. Accurate and robust brain image alignment using boundary-based registration. *Neuroimage*. 2009;48(1):63-72. doi:10.1016/j.neuroimage.2009.06.060

13.	Power JD, Mitra A, Laumann TO, Snyder AZ, Schlaggar BL, Petersen SE. Methods to detect, characterize, and remove motion artifact in resting state fMRI. *Neuroimage*. 2014;84:10.1016/j.neuroimage.2013.08.048. doi:10.1016/j.neuroimage.2013.08.048

14.	Behzadi Y, Restom K, Liau J, Liu TT. A component based noise correction method (CompCor) for BOLD and perfusion based fMRI. *Neuroimage*. 2007;37(1):90-101. doi:10.1016/j.neuroimage.2007.04.042

15.	Satterthwaite TD, Elliott MA, Gerraty RT, et al. An improved framework for confound regression and filtering for control of motion artifact in the preprocessing of resting-state functional connectivity data. *Neuroimage*. 2013;64:240-256. doi:10.1016/j.neuroimage.2012.08.052

16.	Patriat R, Reynolds RC, Birn RM. An improved model of motion-related signal changes in fMRI. *Neuroimage*. 2017;144(Pt A):74-82. doi:10.1016/j.neuroimage.2016.08.051
